# High SARS-CoV-2 tropism and activation of immune cells in the testes of non-vaccinated deceased COVID-19 patients

**DOI:** 10.1186/s12915-022-01497-8

**Published:** 2023-02-16

**Authors:** Guilherme M. J. Costa, Samyra M. S. N. Lacerda, André F. A. Figueiredo, Natália T. Wnuk, Marcos R. G. Brener, Lídia M. Andrade, Gabriel H. Campolina-Silva, Andrea Kauffmann-Zeh, Lucila G. G. Pacifico, Alice F. Versiani, Maísa M. Antunes, Fernanda R. Souza, Geovanni D. Cassali, André L. Caldeira-Brant, Hélio Chiarini-Garcia, Fernanda G. de Souza, Vivian V. Costa, Flavio G. da Fonseca, Maurício L. Nogueira, Guilherme R. F. Campos, Lucas M. Kangussu, Estefânia M. N. Martins, Loudiana M. Antonio, Cintia Bittar, Paula Rahal, Renato S. Aguiar, Bárbara P. Mendes, Marcela S. Procópio, Thiago P. Furtado, Yuri L. Guimaraes, Gustavo B. Menezes, Ana Martinez-Marchal, Kyle E. Orwig, Miguel Brieño-Enríquez, Marcelo H. Furtado

**Affiliations:** 1 Universidade Federal de Minas Gerais, Belo Horizonte, MG Brazil; 2 Clínica MF Fertilidade Masculina, Belo Horizonte, MG Brazil; 3 Faculdade de Medicina de São Jose do Rio Preto, São Jose do Rio Preto, SP Brazil; 4 Department of Pathology, University of Texas Medical Branch, Galveston, TX USA; 5 Department of Obstetrics, Gynecology, and Reproductive Sciences, Magee-Women’s Research Institute, University of Pittsburgh School of Medicine, Pittsburgh, USA; 6 Centro de Desenvolvimento da Tecnologia Nuclear-CDTN/CNEN, Belo Horizonte, MG Brazil; 7 Universidade Estadual Paulista, São José do Rio Preto, SP Brazil; 8 Departamentos de Urologia e de Reprodução Humana da Rede Mater Dei de Saúde, Belo Horizonte, MG Brazil

**Keywords:** SARS-CoV-2 replication, Macrophages, Spermatogonia, Sertoli cell, Leydig cell, Renin-angiotensin system, Infertility, Spermatogenesis, Testosterone, Nanotechnology

## Abstract

**Background:**

Cellular entry of SARS-CoV-2 has been shown to rely on angiotensin-converting enzyme 2 (ACE2) receptors, whose expression in the testis is among the highest in the body. Additionally, the risk of mortality seems higher among male COVID-19 patients, and though much has been published since the first cases of COVID-19, there remain unanswered questions regarding SARS-CoV-2 impact on testes and potential consequences for reproductive health. We investigated testicular alterations in non-vaccinated deceased COVID-19-patients, the precise location of the virus, its replicative activity, and the immune, vascular, and molecular fluctuations involved in the pathogenesis.

**Results:**

We found that SARS-CoV-2 testicular tropism is higher than previously thought and that reliable viral detection in the testis requires sensitive nanosensors or RT-qPCR using a specific methodology. Through an in vitro experiment exposing VERO cells to testicular macerates, we observed viral content in all samples, and the subgenomic RNA’s presence reinforced the replicative activity of SARS-CoV-2 in testes of the severe COVID-19 patients. The cellular structures and viral particles, observed by transmission electron microscopy, indicated that macrophages and spermatogonial cells are the main SARS-CoV-2 lodging sites, where new virions form inside the endoplasmic reticulum Golgi intermediate complex. Moreover, we showed infiltrative infected monocytes migrating into the testicular parenchyma. SARS-CoV-2 maintains its replicative and infective abilities long after the patient’s infection. Further, we demonstrated high levels of angiotensin II and activated immune cells in the testes of deceased patients. The infected testes show thickening of the tunica propria, germ cell apoptosis, Sertoli cell barrier loss, evident hemorrhage, angiogenesis, Leydig cell inhibition, inflammation, and fibrosis.

**Conclusions:**

Our findings indicate that high angiotensin II levels and activation of mast cells and macrophages may be critical for testicular pathogenesis. Importantly, our findings suggest that patients who become critically ill may exhibit severe alterations and harbor the active virus in the testes.

**Supplementary Information:**

The online version contains supplementary material available at 10.1186/s12915-022-01497-8.

## Background

Since the testis displays one of the highest expressions of angiotensin-converting enzyme 2 (ACE2) receptors, which mediate the cellular entry of SARS-CoV-2, and current data suggest that men are more affected than women [1], deep testicular evaluations of patients affected by COVID-19 is imperative. Furthermore, previous studies present discordant results concerning SARS-CoV-2 detection in testicular parenchyma through RT-PCR [2–5] and the extent of testicular damage caused by the virus [5]. Although some testicular alterations promoted by SARS-CoV-2 infection were previously shown [2–5], many of the molecular players of testicular pathogenesis in COVID-19 remain unknown. Indeed, several questions about viral infection remain unexplored, such as the viral replication, route of infection, and the identity of infected cells.

Thus, studying the cellular, enzymatic, hormonal, and critical gene alterations in the testes of non-vaccinated COVID-19 patients should contribute to a better understanding of SARS-CoV-2 biology and its possible impact on testes and male fertility. Recently, Edenfield and Easley [6] published a perspective article in Nature Reviews Urology urging the field to unveil the potential mechanisms involving SARS-CoV-2’s entry and pathophysiological effects on affected subjects.

In this study, we used different methods to detect SARS-CoV-2 in the testis parenchyma of patients deceased with COVID-19 and investigated the virus’s infective and replicative capacities using testicular macerates in VERO cell culture. We also revealed the cellular and molecular alterations in human testicular pathophysiology and correlated the findings with the patient’s clinical data, thus unveiling potential mechanisms underlying the observed alterations.

## Results

### Hypertension, diabetes, and obesity are the main comorbidities

All 11 patients studied herein were admitted to the Intensive Care Unit (ICU) of the Mater Dei Hospital (Belo Horizonte, Brazil) due to severe pulmonary symptoms. The mean age was 63.9 ± 13.11 years (range 46 to 88), and the mean body mass index was 32.18 Kg/m^2^ ± 6.04 (range 25.95–44.46). All patients had children, except for patient #9. Table 1 summarizes the patients’ clinical characteristics. The mean disease duration (from onset to death) was 23.73 ± 8.24 days (range 13–38), while the meantime of ICU admission to death was 15.81 ± 6.19 days (range 8–25). The most prevalent comorbidities were systemic arterial hypertension (nine cases), diabetes mellitus (six cases), and obesity (six cases).

**Table 1:** Clinical features of 11 COVID-19 patients

COVID-19 patient	Age (years)	Body- weight (Kg)	BMI	Smoking habit	Comorbidities	Previous testicular disorder	Fertility record (children)	Disease duration (days)	Hospital stay (days)	Body temp. (max)	Sign of Orchitis	Cause of death
**#1**	75	120	41.5	Yes	SAH, diabetes, obesity	No	Yes	26.00	21.0	38.7 °C	No	Multiple organ failure
**#2**	76	103	31.79	No	SAH, obesity	No	Yes	32.00	25.0	37.9 °C	No	Septic shock
**#3**	56	100	32.65	No	SAH, diabetes, obesity	No	Yes	31.00	20.0	38.4 °C (> 24 h)	No	Septic shock
**#4**	56	80	26.12	No	SAH, diabetes, obesity, hypothyroidism	No	Yes	26.00	16.0	39.3 °C (> 24 h)	No	Multiple organ failure
**#5**	88	88	30.4	No	SAH, diabetes, hypothyroidism, CRI	No	Yes	13.00	10.0	40.2 °C	No	Multiple organ failure
**#6**	76	85	29.41	No	Hypothyroidism	No	Yes	38.00	20.0	38.1 °C	No	Multiple organ failure
**#7**	50	100	34.6	No	SAH, obesity	No	Yes	19.00	10.0	41.5 °C (> 24 h)	No	Septic shock
**#8**	63	75	26.57	No	SAH, diabetes, hypothyroidism, CRI	No	Yes	18.00	11.0	38.4 °C	No	Multiple organ failure
**#9**	62	75	25.95	No	SAH	No	No	14.00	10.0	38.9 °C (> 24 h)	No	Multiple organ failure
**#10**	55	130	44.46	No	SAH, diabetes, obesity	No	Yes	28.00	23.0	37.9 °C (> 24 h)	No	Multiple organ failure
**#11**	46	99	30.56	No	No	No	Yes	16.00	8.0	40.9 °C (> 24 h)	No	Multiple organ failure

*BMI* body mass index, *SAH* systemic arterial hypertension, *CRI* chronic renal insufficiency, *temp.* temperature

### SARS-CoV-2 reliable detection in testes

We first tested the testicular tissue using a conventional RT-qPCR protocol for SARS-CoV-2, but only patient #8 was positively detected (cycle threshold—CT = 38). To improve detection and reduce interference of intrinsic tissue factors, we performed a cDNA synthesis using SARS-CoV-2 specific viral primers. The RT-qPCR revealed the virus’s presence in 10 of 11 patients (Table 2).

**Table 2:** SARS-CoV-2 detection in testicular parenchyma

	Genetic evaluations				Protein evaluations			Infection of VERO cells					
Patients	E gene (RD) (CT)	RNP ctrl (CT)	N1 gene (SP) (CT)	β-actin ctrl (CT)	LSPR-nanosensor N shift (nm)	LSPR-nanosensor S shift (nm)	IF Spike (−/+)	1st day- N1 gene (CT)	2nd day- N1 gene (CT)	RNP ctrl (CT)	+ RNA (CT)	− RNA (CT)	IF Spike (−/+)
**CTRL #1**	-	20	-	18.55	0 (−)	0 (−)	-	-	-	20	-	-	-
**CV-19 #1**	-	23	32.78 (+)	24.59	31.5 (+)	51 (+)	+	34 (+)	33 (+)	29	29 (+)	28 (+)	+
**CV-19 #2**	-	27	36.99 (+)	18.50	13.2 (+)	18.9 (+)	+	34 (+)	34 (+)	27	32 (+)	33 (+)	-
**CV-19 #3**	-	22	36.34 (+)	6.36	1.2 (−)	17.5 (+)	+	34 (+)	31 (+)	30	26 (+)	25 (+)	+
**CV-19 #4**	-	20	35.96 (+)	19.25	11.2 (+)	17 (+)	+	34 (+)	32 (+)	29	29 (+)	28 (+)	+
**CV-19 #5**	-	20	36.14 (+)	20.03	9.9 (+)	20.9 (+)	+	32 (+)	27 (+)	32	23 (+)	25 (+)	+
**CV-19 #6**	-	21	36.22 (+)	10.30	13.2 (+)	15 (+)	+	34 (+)	32 (+)	29	27 (+)	27 (+)	-
**CV-19 #7**	-	20	35.37 (+)	17.67	4.5 (−)	12.4 (+)	+	34 (+)	32 (+)	25	-	-	+
**CV-19 #8**	38 (+)	20	35.95 (+)	19.34	17.5 (+)	17 (+)	+	34 (+)	34 (+)	26	-	-	+
**CV-19 #9**	-	21	-	19.81	12.9 (+)	17.5 (+)	+	35 (+)	34 (+)	27	32 (+)	35 (+)	-
**CV-19 #10**	-	23	34.03 (+)	20.71	7.2 (+)	13.6 (+)	+	34 (+)	32 (+)	30	-	-	-
**CV-19 #11**	-	23	35.65 (+)	16.27	8.7 (+)	16.5 (+)	+	34 (+)	33 (+)	30	29 (+)	33 (+)	-

*CV-19* COVID-19 patient, *RNP* RNAse P gene, *RD* routinary diagnosis, *SP* specific primers, *CT* cycle threshold, *LSPR* localized surface plasmon resonance, *IF* immunofluorescence, *Spike* spike protein; *(+)*, detection of viral content; *(-)*, non-detection of viral content. Positivity in the RT-qPCR is detected when the CT is lower than 40, and in the LSPR nanosensor is when the shift is higher than 5 nm

To confirm the genetic data, we used a nano-designed sensor, which employs localized surface plasmon resonance—LSPR [7], to detect the SARS-CoV-2 Spike (S)-protein and the nucleocapsid (N)-protein (Additional file 1: Fig. S1a-q). S-protein was found in all patients, while N-protein was observed in nine patients (Table 2 and Additional file 1: Fig. S1m-q). In addition, prominent S-protein immunolabeling was evidenced in the testes of all COVID-19 patients (Additional file 1: Fig. S2a-p), especially in patient #8 (positively detected by all methodologies). Our data suggest that more sensitive techniques are required for the reliable detection of SARS-CoV-2 (even in a low viral titer) in testes.

### Main infected cells in testis parenchyma

**Fig. 1:**
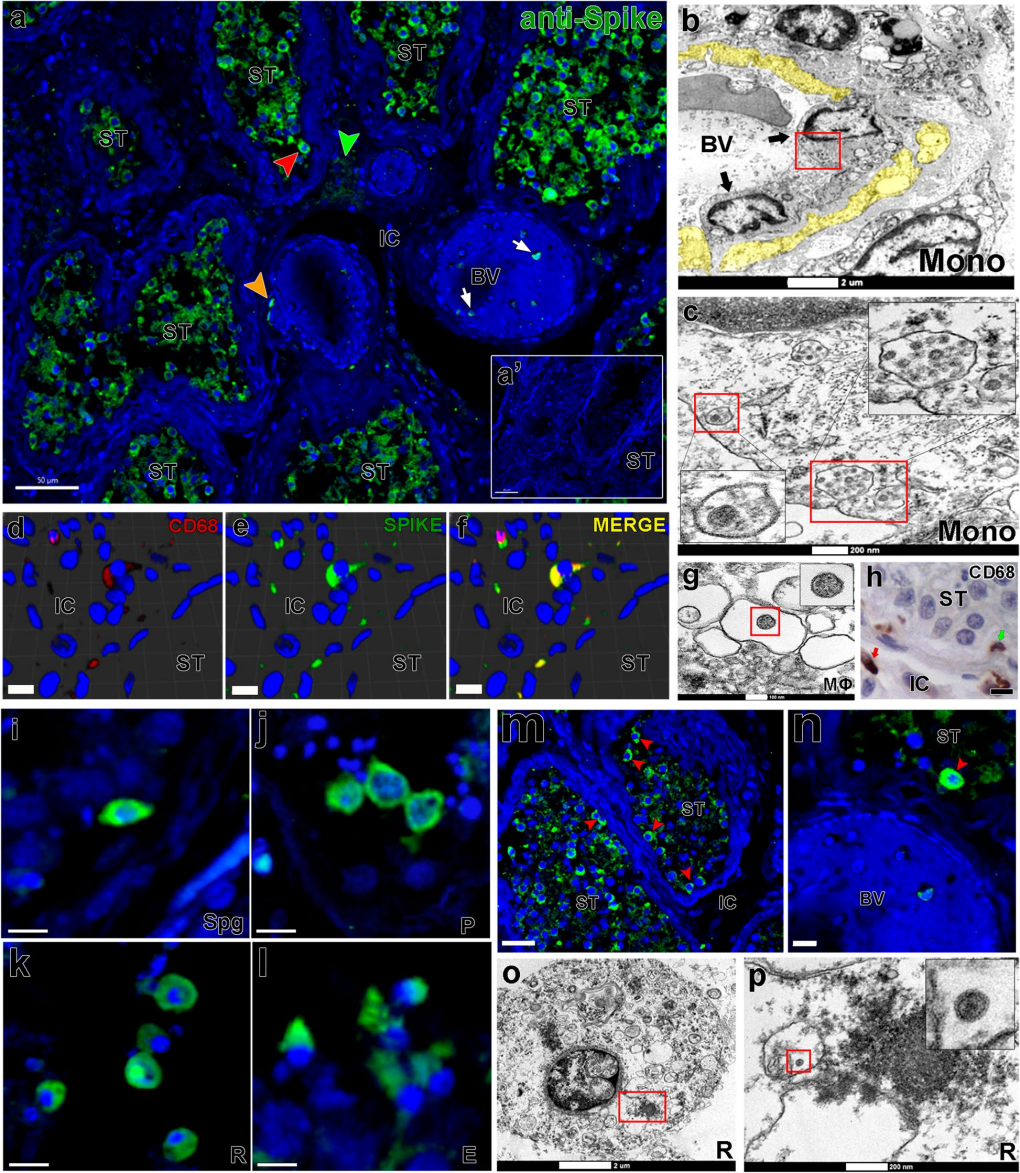
SARS-CoV-2 infection in monocytes, macrophages, and germ cells. **a** Immunofluorescence for S-protein in testicular parenchyma showing positive germ cells (red arrowhead), monocytes (white arrow), macrophages (orange arrowhead), and Leydig cells (green arrowhead); **a’** negative control omitting the primary antibody (scale bars = **a** 50 μm; **a’** 40 μm). **b**, **c** TEM images showing infected infiltrative monocytes (mono, arrows); yellow = disrupted cytoplasm of endothelial cells; red boxes = cytoplasmic areas replenished with viral particles; inserts = high magnification of viral particles (Scale bars = **g** 2 μm; **h** 200nm). **d**–**f** 3D reconstruction of double immunofluorescence for CD68 (w, red color) and S-protein (x, green color), depicting an infected macrophage (y, merge–yellow color) (scale bars = 10μm). **g** TEM image of an infected macrophage (m) showing a viral particle in its cytoplasm (insert). **h** Presence of macrophages (arrows) inside the seminiferous tubule (green arrow) and in the interstitial area (red arrow) (scale bar = 10μm). **i**–**l** Different types of infected germ cells: **i** spermatogonial cell (Spg); **j** pachytene spermatocytes (P); **k** round spermatids (R); **l** elongated spermatids (E) (scale bars = 15μm). **m**–**n** Immunofluorescence against the S-protein shows that spermatogonial cells (red arrowheads) are more intensely labeled than other germ cells (scale bars = **a** 30 μm; **b** 10 μm). **o**–**p** TEM images showing viral particles (in low and high magnification) in round spermatids (scale bars = **o** 2 μm; **p** 200 nm). Immunofluorescence images in the testis of patient #8. TEM images in testes from patients #1, #7, and #8. Blue = dapi counterstaining; green staining = IgG-CFL 488; red staining = IgG- 546. ST, seminiferous tubule cross-section; IC, intertubular compartment; BV, blood vessel

Several infected monocytes/macrophages (CD68+) were detected surrounding blood vessels and migrating to the parenchyma (IF and TEM data), suggesting that these cells might be delivering SARS-CoV-2 to the testes (Fig. 1a–c, arrows), and contributing to the infection of testicular cells. Infected macrophages were confirmed through double immunofluorescence and transmission electron microscopy (TEM) (Fig. 1d–g). Monocytes/macrophages were also found inside the tubular compartment, indicating a possible route for viral spreading inside the seminiferous tubules (Fig. 1h). Conversely, chymase-positive mast cells were not labeled for S-protein (Additional file 1: Fig. S3a-b).

Most S-protein labeling was identified inside the seminiferous tubules, mainly in germ cells (Fig. 1i–m). In some areas, spermatogonial cells displayed intense S-protein labeling compared to spermatocytes and spermatids (Fig. 1n, red arrowhead). SARS-CoV-2 was detected in different types of germ cells (Fig. 1o–p). Patients who died up to 20 days after ICU admission presented the highest mean fluorescence index in the testis (Additional file 1: Fig. S2a).

**Fig. 2:**
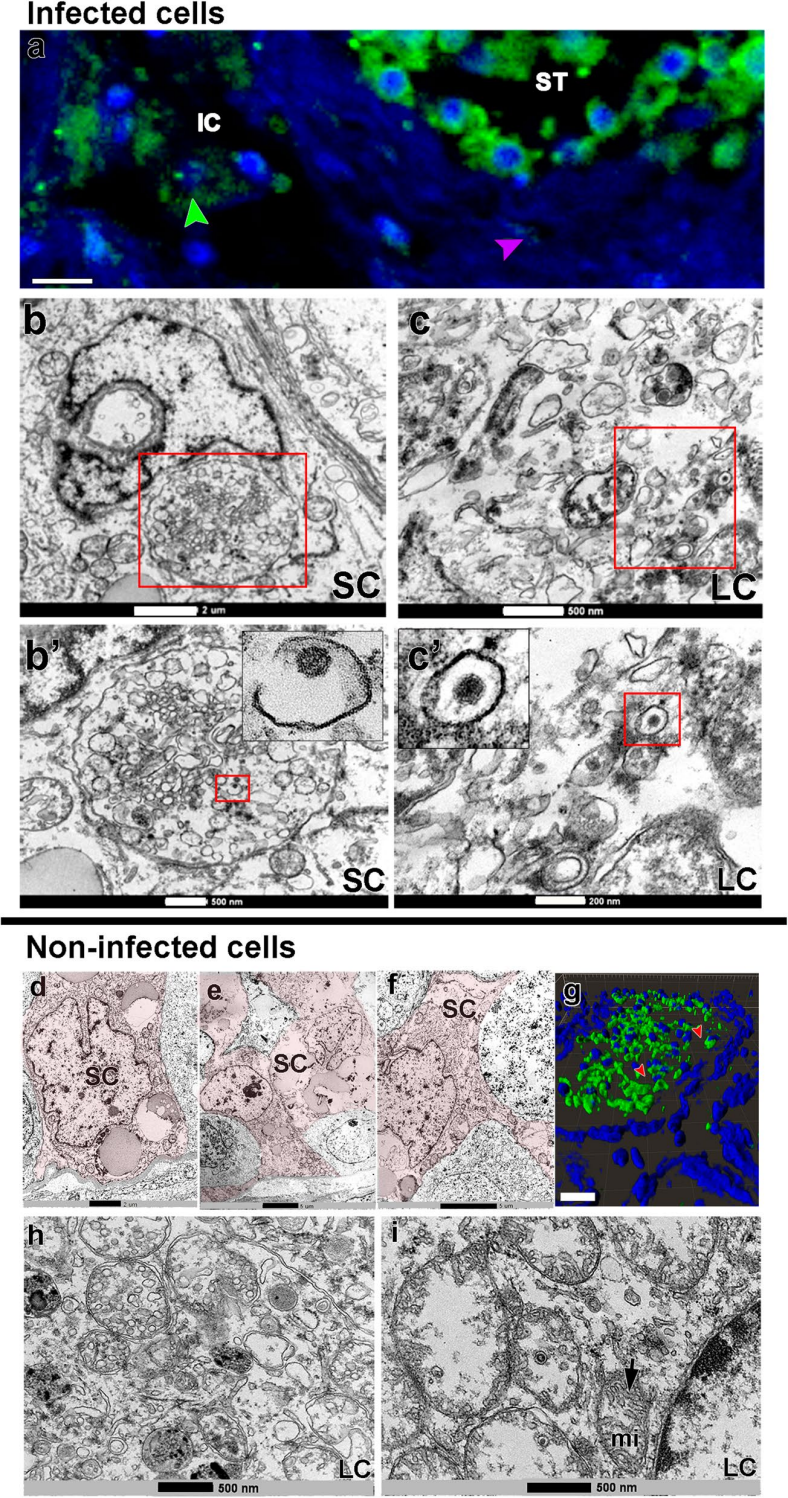
SARS-CoV-2 infection in testicular somatic cells. **a** Immunofluorescence against S-protein evidencing weak labeling in peritubular myoid (pink arrowhead) and Leydig cells (green arrowhead) (scale bar =15 μm). **b**–**c’** TEM images showing viral particles (in low and high magnification) in the Sertoli cell (SC) (scale bars = **b** 2 μm; **b'** 500 nm) and Leydig cell (LC) (scale bars = **c** 500 nm; **c'** 200 nm). **d**–**f** TEM images of non-infected Sertoli cells (SC, pink) (scale bars = **e** 2 μm; **f**–**g** 5μm). **g** 3D reconstruction of a seminiferous tubule cross-section showing non-labeled areas surrounding germ cells (red arrowheads) (Scale bar = 40 μm). **h**–**i** high magnification of non-infected Leydig cells. Arrow = tubular crest of a mitochondrion (mi) (scale bars = 500 nm). Immunofluorescence images in the testis of patient #8. TEM images in testes from patients #1, #7, and #8

Some Sertoli and Leydig cells presented viral particles in their cytoplasm (TEM data), albeit with lower S-protein immunolabeling intensity (Fig. 2a-c’, arrowheads and insets). However, many of these cells did not show obvious viral particles in the cytoplasm (TEM data) (Fig. 2d–i).

### Viral replication and viability

**Fig. 3:**
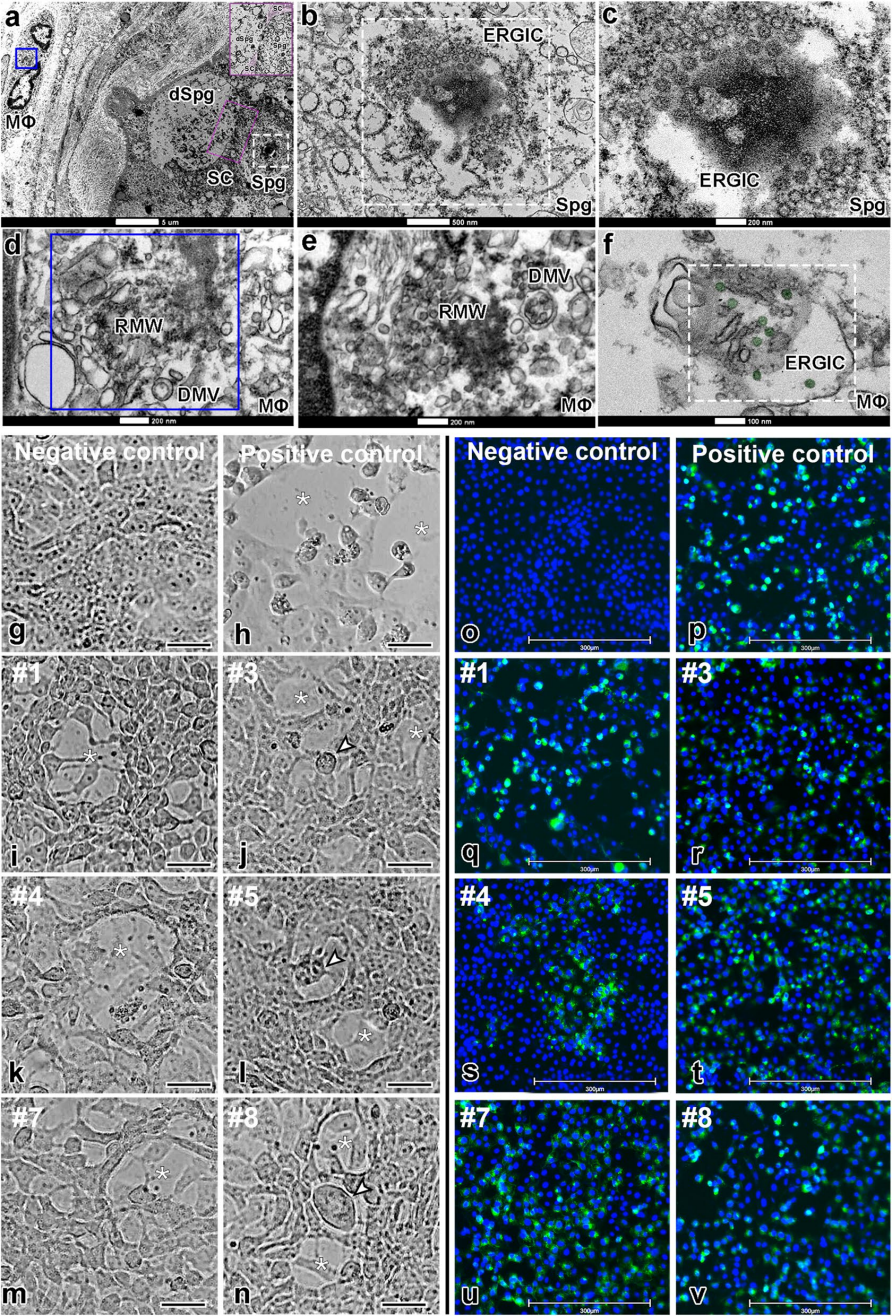
Infective SARS-CoV-2 particle formation and replication activity in Vero cells. **a**–**f** TEM images of testicular parenchyma (scale bars = **a** 5 μm; **b** 500 nm; **c** 200 nm; **d** 200 nm; **e** 200 nm; **f** 100 nm). **a** Altered seminiferous tubule cross-section, depicting a macrophage in tunica propria containing a replication membranous web (RMW; blue box) and a spermatogonial cell displaying an endoplasmic reticulum Golgi intermediate complex (ERGIC, white box). The pink box indicates a spermatogonial cell clone surrounded by the cytoplasm of a Sertoli cell (labeled in pink). **b**, **c** Higher magnifications of spermatogonial cells showing structures similar to new virions inside the ERGIC. Although very large, this structure also resembles a hijacked nuclear pore complex (present in situations of viral replication). **d** Higher magnification of the RMW from the macrophage depicted in “figure a,” evidencing the presence of double-membrane vesicles (DMV). **e**–**f** Images from an interstitial macrophage presenting RMW, DMV, and ERGIC filled with new virions (labeled in green). **g**–**n** The cytopathic effect of SARS-CoV-2 was evaluated in VERO CCL-81 cells (empty area, *). Scale bars = 30 μm. **g** Cell confluence of VERO CCL-81 cultures before exposition to testicular macerates. **h** VERO CCL-81 cells exposed to SARS-CoV-2 (positive control). **i**–**n** VERO CCL-81 cells exposed to testicular macerates of COVID-19 patients (empty area, *) (arrowheads indicate structures similar to a syncytium cell). Three wells were replicated per patient. **o**–**v** S-protein immunofluorescence in VERO CCL-81 cells (blue labeling: DAPI; green labeling: spike protein). Scale bars = 300 μm. **o** Negative control omitting the primary antibody. **p** Positive control using VERO CCL-81 cells infected with SARS-CoV-2. **q**–**v** Immunofluorescence in VERO CCL-81 cell culture after exposition to testicular homogenates

TEM analyses identified a viral replication factory in macrophages and spermatogonial cells (Fig. 3a–f). In both cell types, we observed SARS-CoV-2 replication complexes with the formation of convoluted membranes (replication membranous webs, RMW) containing double-membrane vesicles (DMV) and endoplasmic reticulum Golgi intermediate complex (ERGIC) showing new virions (Fig. 3c–f).

In a biosafety level-3 (BSL-3) lab, we exposed VERO CCL-81 cell cultures to testicular homogenates from all patients (Fig. 3g–v). We found that SARS-CoV-2 was infective and replicating in these cells, diminishing the CT value (N1 gene) along with the culture time (Table 2). The cytopathic effect (patients #1, #3, #4, #5, #7, and #8; Fig. 3g–n), immunofluorescence (patients #1, #3, #4, #5, #7, and #8; Fig. 3o–v), immunoperoxidase (patient #1; Additional file 1: Fig. S2q-s), RT-qPCR (genes N1) (all samples; Table 2), and identification of the genomic and subgenomic SARS-CoV-2 RNA (patients #1, #2, #3, #4, #5, #6, #9, and #11; Table 2) in the infected VERO cell culture confirmed the viral replication. SARS-CoV-2 was detected in the testes of all patients who died either soon or long after the onset of symptoms. The replicative data indicate we were not observing just fragments of viral particles. Our data also suggest that the virus remains viable within the testis of a severe COVID-19 patient for an extended period.

### Clinical data and testicular alterations

**Fig. 4:**
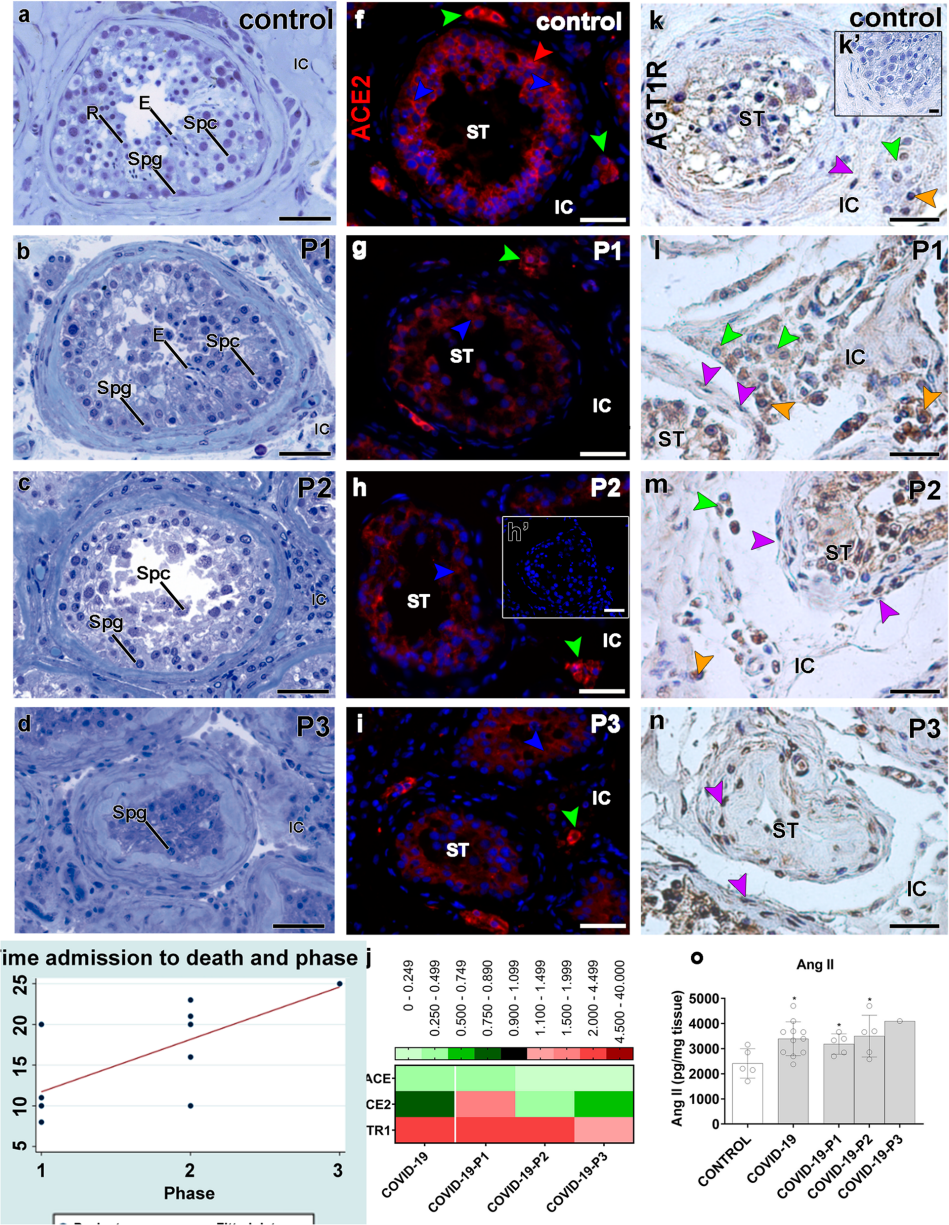
Imbalanced renin-angiotensin system in the testis of COVID-19 critically ill patients. **a**–**d** Initial categorization of COVID-19 patients according to the most advanced germ cell in the seminiferous epithelium: phase 1 (P1), phase 2 (P2), and phase 3 (P3). Toluidine-blue counterstaining (scale bars = 50 μm). **a** Control patients showing elongated spermatids (E). **b** COVID-19 patients showing elongated spermatids (E), spermatocytes (Spc), and spermatogonial cells (Spg). **c** COVID-19 patients displaying spermatocytes (Spc) and spermatogonial cells (Spg). **d** COVID-19 patients presenting spermatogonial cells (Spg) only. **e** Positive correlation between patient’s phases and the time frame from ICU admission to death (Spearman’s rho = 0.6862; *p* = 0.0197). **f**–**i** Immunostaining of ACE2 in the testis of control (**f**) and COVID-19 patients (**g**–**i**) (scale bars = 50 μm). **h’** Negative control, omitting the primary antibody. Red: Alexa-594; blue: dapi; green arrowheads: Leydig cell; red arrowheads: spermatogonial cell; blue arrowheads: Sertoli cell. **j** Heat map of the evaluated genes related to the renin-angiotensin system. The colors compare COVID-19 patients to controls. Green: lower expression than controls. Red: higher expression than controls. **k**–**n** Immunostaining of AGT1R in the testis of control (**k**) and COVID-19 patients (**l**–**n**). Purple arrowheads: peritubular myoid cells; green arrowheads: Leydig cells; orange arrowhead: macrophages (scale bars = 50 μm). **o** ELISA assay for angiotensin II (*t*-test; two-tailed; infected groups vs control; *p < 0.05). P1, P2, P3: phases 1, 2, and 3 of the COVID-19 patients, respectively. ST, seminiferous tubule; IC, intertubular compartment. Individual values can be found at 10.6084/m9.figshare.16777786.v8

Although we presented the data of all patients in a single group (COVID-19), the COVID-19 patients were further subdivided into groups according to the phenotype of the testes’ parenchyma. Critically ill patients who maintained elongated spermatids in the seminiferous epithelium were categorized as phase I patients (patients #6, #7, #8, #9, and #11). Patients presenting primary spermatocytes as the most advanced germ cell type were categorized as phase II patients (patients #1, #3, #4, #5, and #10). Specifically, patient #2 was classified as phase III because his testis presented few spermatogonia inside the seminiferous tubules (Fig. 4a–d). A weak correlation (Spearman’s = − 0.33; *p* = 0.30) was observed between the germ cell loss and the patient’s age. Furthermore, we did not observe a strong correlation between the patient’s age and the progression of the pathogeny (Spearman’s = 0.33; *p* = 0.31).

Remarkably, there was a positive correlation (Spearman’s = 0.6862; *p* = 0.0197) between the above-described phases and the time from ICU admission to death (Fig. 4e). All phase I patients died less than 20 days after ICU admission, with most deceasing well before 20 days. All phase II patients died 20–23 days after ICU admission. Patient #2 (phase III) died after 25 days. Our findings suggest that the more extended the severe condition (ICU stay), the lower the number of surviving germ cells. It is essential to mention that none of the patients presented scrotal symptoms or complaints during their hospital stay prior to ICU admission. Also, the clinical history did not reveal previous testicular disorders. All patients presented fever during hospitalization, six of them for longer than 24 h. However, we did not find evidence of a strong correlation between fever and the development of testicular pathogenesis (Spearman’s = − 0.195; *p* = 0.57).

### Imbalanced renin-angiotensin system

We found ACE2 protein in spermatogonial, Leydig, and Sertoli cells of Controls and COVID-19 patient testes (Fig. 4f). However, testicular cells of COVID-19 patients showed weak ACE2 staining intensity, especially inside the seminiferous tubules (Fig. 4g–i). qPCR revealed augmented ACE2 expression in the testis of phase I patients and diminished in Phase II and III patients (Fig. 4j). ACE protein expression reduced, whereas angiotensin II receptor type 1 (AGT1R) increased in all phases (Fig. 4j).

COVID-19 patients presented more intensely immunolabeled testicular cells for the AGT1R than controls, particularly in Leydig cells, macrophages, and peritubular myoid cells (Fig. 4k–n). Angiotensin II levels were higher in the testis parenchyma of COVID-19 patients than in Controls (Fig. 4o).

### Mast cell and macrophage activation

**Fig. 5:**
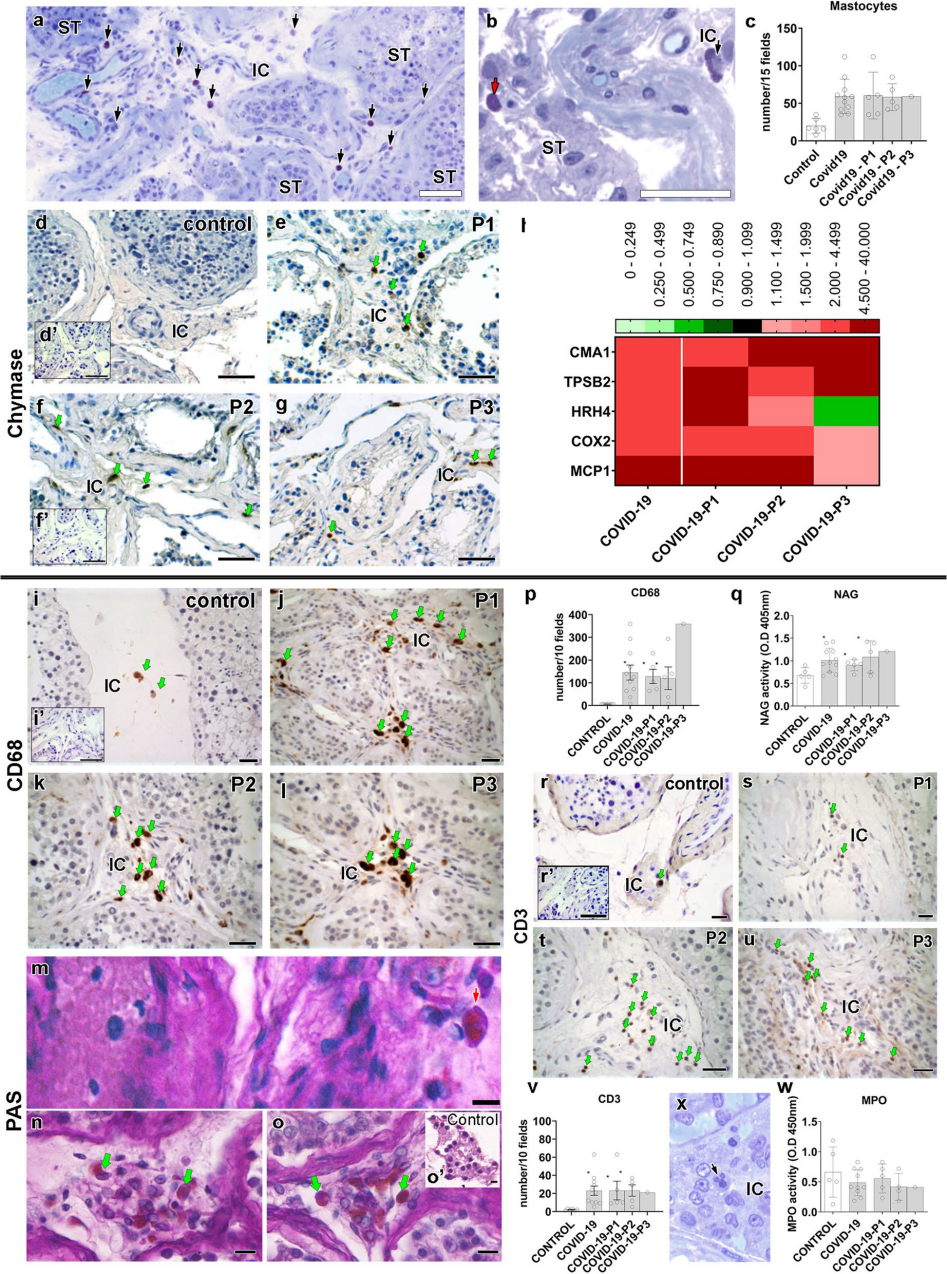
Presence of activated mast cells and macrophages in the testis parenchyma of COVID-19 critically ill patients. **a** Toluidine blue staining showing an elevated number of mast cells (arrows) in testis parenchyma (scale bar = 50 μm). **b** In some regions, mast cells were identified inside the seminiferous tubules (red arrow). **c** Number of mast cells counted in 15 fields of testis parenchyma at × 400 magnification (*t*-test; two-tailed; infected groups vs control; *p < 0.05). **d**–**g** Immunostaining for chymase in testes of control and COVID-19 patients. Activated mast cells (arrows) were observed in COVID-19 patients only (scale bars = 50 μm). **d’** Negative control (scale bars = 50 μm). **h** Heat map of the evaluated genes related to the mast cells and macrophages. The colors compare COVID-19 patients to controls. Green: lower expression than controls. Red: higher expression than controls. **i**–**l** Immunostaining for CD68 in testes of control and COVID-19 patients, illustrating the high prevalence of this cell in the deceased patients (scale bars = 50 μm). **i’** Negative control (scale bars = 50 μm). **m**–**o** PAS-positive monocytes (red arrow) and macrophage (green arrow) indicating their activation (scale bars = 15 μm). **o’** PAS-negative cells in the interstitial cells of a control patient (scale bar = 10 μm). **p** Number of macrophages counted in 10 fields of testis parenchyma at × 400 magnification (*t*-test; two-tailed; infected groups vs control; *p < 0.05). **q** N-acetylglucosaminidase (NAG) assay indicating the activation of macrophages in COVID-19 patients’ testes (*t*-test; two-tailed; control vs infected group; *p* > 0.05). **r**–**v** Immunostaining for CD3 and lymphocyte number in testes of control and COVID-19 patients (*t*-test; two-tailed; infected groups vs control; *p < 0.05) (scale bars = 50 μm). **r’** Negative control (scale bars = 50 μm). **x**–**w** Neutrophils and their activity (s: MPO assay) in testis parenchyma (*t*-test; two-tailed; control vs infected group; *p* > 0.05) (scale bar = 10 μm). Individual values can be found at 10.6084/m9.figshare.16777786.v8

Total protein was significantly higher in testes from COVID-19 patients than in controls (Additional file 1: Fig. S3c). Our morphological and molecular data suggest that mast cells and macrophages play a critical role in testicular pathogenesis. The number of mast cells found in the testis of COVID-19 patients was ten times higher than that found in controls (Fig. 5a–c). Immunohistochemistry revealed chymase-positive mast cells in the testes of the COVID-19-affected patients (Fig. 5d–g). Interestingly, mast cells were detected next to areas of the testicular parenchyma displaying a high concentration of immune cells, hemorrhagic areas, altered Leydig cells, interstitial fibrotic areas, damaged tunica propria, and thickened tubular basal membrane (Additional file 1: Fig. S3d-m). qPCR data showed that Control testes presented minimal expression of chymase (CMA1) and tryptase (TPSB2), whereas all testicular samples from the COVID-19 patients exhibited increased expression (Fig. 5h). In phase I patients, mast cells were highly prevalent in the testes’ intertubular compartment. Phase II and III patients showed these cells inside the tubular and intertubular compartments (Additional file 1: Fig. S3j-m).

Testes of COVID-19 patients also presented elevated mRNA levels of histamine H4 receptor (HRH4), cyclooxygenase-2 (COX2) and monocyte chemoattractant protein-1 (MCP1; 10x higher) (Fig. 5h). MCP1 is particularly unfavorable for testicular pathogenesis because it can attract more infected monocytes (Additional file 1: Fig. S3n-o). Macrophage (CD68+) numbers were augmented, and these cells were highly active (N-acetyl-BD-glucosaminidase (NAG) and periodic acid–Schiff (PAS) assays) in infected testis (Fig. 5i–q).

Taken together, our findings suggest a significant contribution of activated mast cells and macrophages to the pathophysiology of COVID-19 in the human testes, which could eventually induce harmful effects on the reproductive health of severely ill patients.

Lymphocytes (CD3+) were frequently observed in the testes of COVID-19 patients, suggesting a prolonged infection (Fig. 5r–v). Neutrophils were also identified in the testes of COVID-19 patients (Fig. 5x); however, their myeloperoxidase activity (MPO assay) was not altered when compared to Controls (Fig. 5w). These data suggest that macrophages are more active than neutrophils in the testes of COVID-19 patients.

### Effects on the tubular compartment

While the germ cell population and tubular diameter diminished in COVID-19 patients (Additional file 1: Fig. S4a-b), the tunica propria enlarged, reflecting a higher number of peritubular myoid cells and collagen fibers (Additional file 1: Fig. S4c-j). Interestingly, the tunica propria and the basal membrane (PAS+) thickened next to mast cells and macrophages and sometimes presented a corkscrew appearance (Additional file 1: Fig. S4k-n).

**Fig. 6:**
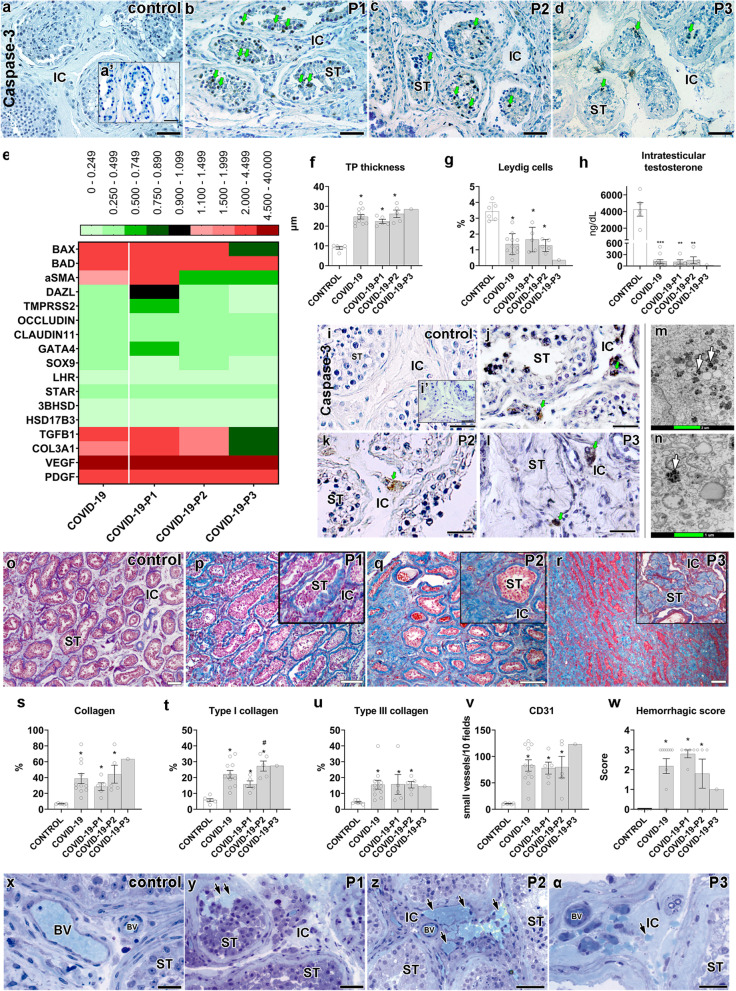
Main testicular dysfunctions following SARS-CoV-2 infection. **a**–**d** Immunostaining showing Caspase-3 positive germ cells (green arrows) in testes of controls and COVID-19 patients (scale bars = 50 μm). **a’** Negative control (scale bars = 50 μm). **e** Heat map of critical genes from the tubular and intertubular compartments. The colors compare COVID-19 patients to controls. Green: lower expression than controls. Red: higher expression than controls. **f** Measurement of the tunica propria thickness (*t*-test; two-tailed; infected groups vs control; *p < 0.05). **g** Leydig cell volume density in testes of controls and COVID-19 patients (*t*-test; two-tailed; infected groups vs control; *p < 0.05). **h** Measurement of intratesticular testosterone using RIA (*t*-test; two-tailed; infected groups vs control; ***p < 0.001; **p < 0.01). **i**–**l** Immunostaining for caspase 3, evidencing positive labeling in Leydig cells (green arrows) (scale bars = 50 μm). **i’** Negative control (scale bars = 50 μm). **m**–**n** TEM showing the presence of several electron-dense vacuoles in Leydig cell cytoplasm (white arrows) (scale bars = **m** 2 μm; **n** 1 μm). **o**–**r** Trichrome Masson staining in the testis of controls and COVID-19 patients. In higher magnification, inserts (P1, P2, and P3) show the collagen deposition in the seminiferous tubules (scale bars = 200 μm). **s**–**u** Quantification of total collagen fibers, type I collagen, and type III collagen (*t*-test; two-tailed; infected groups vs control; *p < 0.05; COVID-19-P1 vs COVID-19-P2; #*p* < 0.05). **v** Quantification of newly formed blood vessels (CD31+) (*t*-test; two-tailed; infected groups vs control, *p < 0.05). **w** Quantification of testicular hemorrhage (*t*-test; two-tailed; infected groups vs control, *p < 0.05). Testicular hemorrhage was classified as normal (**x**) and as containing high numbers of red blood cells (arrows) in tubular and intertubular compartments (**y**), a vast number of red blood cells in the intertubular compartment (**z**), and few red blood cells in the intertubular compartment (**α**) (scale bars = 50 μm). P1, P2, P3: phases 1, 2, and 3 COVID-19 patients, respectively. BV, blood vessels; IC, intertubular compartment; ST, seminiferous tubule cross-section. Individual values can be found at 10.6084/m9.figshare.16777786.v8

There were many caspase-3-positive germ cells in the testes of all COVID-19 patients (Fig. 6a–d). The rete testis area presented degenerating germ cells (Additional file 1: Fig. S4o), and BAD and BAX levels were augmented in nearly all COVID-19 patients (Fig. 6e). DAZL and TMPRSS2 genes, known to be highly expressed in germ cells, diminished as the phases progressed (Fig. 6e).

Tight junctions of the seminiferous tubules were also compromised in COVID-19 patients, with reduced levels of occludin and claudin-11 (Fig. 6e). Expressions of critical Sertoli cell genes (Sox9 and Gata4) were also reduced (Fig. 6e). Moreover, the seminiferous epithelium detached from the thickened tunica propria (Fig. 6f and Additional file 1: Fig. S4p-t), indicating downregulation of tubular junctional proteins.

### Leydig cells apoptosis and inhibition

There was a gradual volumetric reduction of Leydig cells (Fig. 6g) as the testicular pathogeny developed. Intratesticular testosterone levels measured in the testicular homogenates of COVID-19 patients were lower (~30 times) than the controls (Fig. 6h). Caspase-3 immunolabeling revealed that these cells were undergoing apoptosis (Fig. 6i–l). Additionally, their morphology was altered, presenting a vacuolated cytoplasm (with a degenerating content) (Fig. 6m–n and Additional file 1: Fig. S5a-d).

Reduction in the expression of LHR, STAR, 3BHSD, and 17BHSD in the testis of COVID-19 patients was consistent with the observed low number of Leydig cells (Fig. 6e). The levels of HRH4 were highly augmented in the affected patients (Fig. 5h). This data indicates that local histamine can inhibit steroidogenesis in the testes of COVID-19 patients.

### Testis fibrosis and vascular alterations

Testicular fibrosis was commonly observed in the testis parenchyma of COVID-19 patients. Collagen fibers increased progressively (Fig. 6o–u), showing high amounts of type I and III collagen in testis parenchyma (Fig. 6t–u, Additional file 1: Fig. S5e-h). Further, the expression levels of Col3a were highly augmented in the testes of phase I patients (Fig. 6e).

Expressions of the angiogenic factors VEGF and PDGFA and small vessel volumetric proportion (CD31+) increased in the testis of COVID-19 patients (Fig. 6e). Immature blood vessels (TEM and Immunohistochemistry data; CD31+ cells) were observed inside the tunica propria, suggesting an angiogenic process (Fig. 6v and Additional file 1: Fig. S5i-n). Red blood cells outside the testicular vasculature were frequently observed in the testes of COVID-19 patients (Fig. 6w–α, arrows). Phase I patients presented many red blood cells inside the seminiferous tubule lumen and intratesticular rete testis, possibly due to a reduction in tight epithelial junctions (Additional file 1: Fig. S5o). Some thrombi were detected inside the vascular system (Additional file 1: Fig. S5p).

Furthermore, Endothelin 1/2/3, a protein involved in blood-vessel vasoconstriction, was highly expressed in the first phase patients but reduced in others (Additional file 1: Fig. S5q-t).

## Discussion

**Fig. 7:**
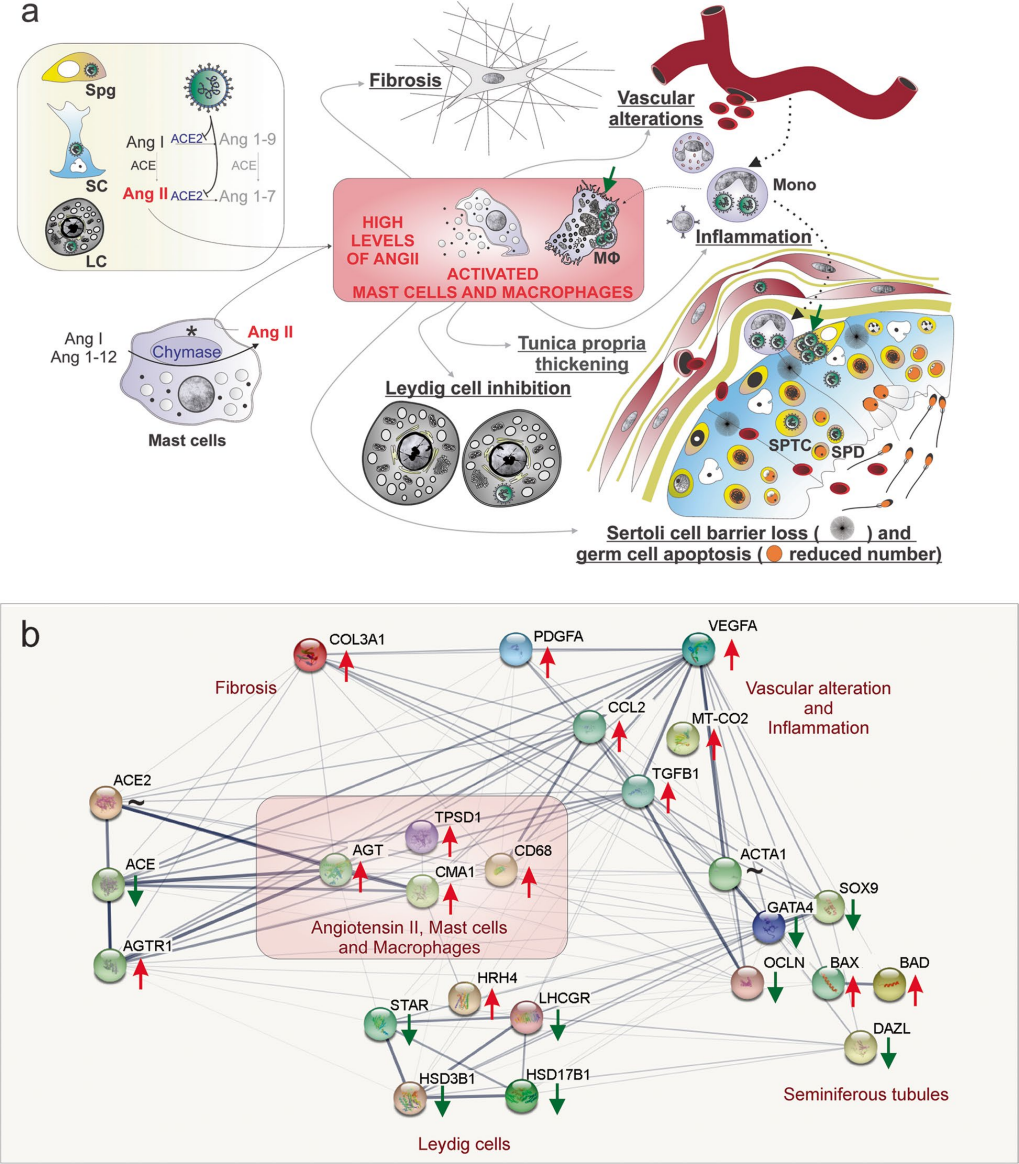
Hypothetical viral and molecular mechanisms of testis infection and damage by SARS-CoV-2. **a** SARS-CoV-2 (green color) was identified in spermatogonial cells (Spg), Sertoli cells (SC), Leydig cells (LC), infiltrative monocytes (Mono), macrophages (MΦ), spermatocytes (sptc), and spermatids (sptd). Note viral factories in macrophages and spermatogonial cells (green arrows). A direct influence of SARS-CoV-2 in testicular cells hampers ACE2 activity, while activation of mast cells (chymase positive) elevates the levels of angiotensin II (a potent pro-inflammatory molecule) (asterisks). Angiogenic and inflammatory factors can induce the infiltration and activation of mast cells. High levels of angiotensin II, activation of mast cells, and inflammatory factors can activate (polarize) macrophages. The testicular phenotype of COVID-19 patients (fibrosis, vascular alteration, inflammation, tunica propria thickening, Sertoli cell barrier loss, germ cell apoptosis, and inhibition of Leydig cells) can be linked to elevated angiotensin II and active mast cells and macrophages. **b** Genes network related to angiotensin II, activated mast cells, and macrophages (pink box) extracted from STRING (https://string-db.org/). These three elements upregulate the inflammatory, apoptotic, fibrotic, and vascular genes while downregulating critical seminiferous tubule and Leydig cell genes. Red arrows: upregulated genes; green arrows: downregulated genes; ~: genes upregulated and downregulated depending on the phase

Herein, we used different and sensitive methods to detect SARS-CoV-2 proteins, genomic and subgenomic RNA, and virus particles in the testis of deceased patients with COVID-19. In Fig. 7, we hypothesized the potential viral, cellular, and molecular mechanisms of infection, replication, and damage by SARS-CoV-2 in the testes of non-vaccinated patients who became severely ill, admitted to the ICU, and eventually died from the disease. Our data suggest a direct influence of SARS-CoV-2 in testicular cells, which might probably deregulate ACE2, elevating the levels of angiotensin II, a potent pro-inflammatory and angiogenic peptide. Consequently, angiogenic and inflammatory factors might induce the infiltration and activation of mast cells and macrophages in the testes of COVID-19 patients. Taken together, the deleterious effects observed in the testes of COVID-19 patients, i.e., fibrosis, vascular alteration, inflammation, tunica propria thickening, Sertoli cell barrier loss, germ cell apoptosis, and inhibition of Leydig cells, seem to be associated with elevated angiotensin II and activation of mast cells and macrophages (Fig. 7a). A protein-protein (or gene-gene) network (Fig. 7b) shows the interaction of factors associated with angiotensin II regulation, activation of mast cells and macrophages, and alterations in testes tubular and intertubular compartments.

Six methodologies, including two types of RT-qPCR, immunofluorescence, Vero cell culture, nanosensors, and TEM were used to clarify and evidence the presence of SARS-CoV-2 in the testes. These findings are critical because previous studies presented conflicting results regarding the detection of SARS-CoV-2 RNA in the testes [2–5]. The data also suggest that SARS-CoV-2 tropism for testes is higher than previously thought and that the conventional RT-qPCR protocol may only detect infected testes with higher viral loads. The precise detection of nanosensors may be related to their capacity to detect femtograms of viral proteins, as shown in the present study. One can say that better viral genetic detection in VERO cell culture may be related to the enrichment of SARS-CoV-2 in the cell culture supernatant. An experimental study in monkeys revealed that SARS-CoV-2 have high tropism to the testes and that the viral signal was higher in the gonads than in the lung [8]. Accordingly, we observed many germ cells positive for the S-protein, and other authors also detected the N-protein in germ cells [4]. Our finding is reinforced by the detection of SARS-CoV-2 RNA in the semen of patients who suffered severe forms of COVID-19 [9]. However, further studies should be undertaken to examine this possibility thoroughly. In a systematic review [10], the authors indicated the low risk of SARS-CoV-2 detection in the semen of recovered men after collecting data from different studies.

We detected infected infiltrating monocytes in deceased patients with severe COVID-19 (non-vaccinated). It is possible that the “macrophage paradox” described for SARS-CoV-2-induced lung damages [11] may also apply to the testes. Thus, although macrophages combat viral infections, they can also act as “Trojan horses” [11], facilitating viral entrance and replication of the virus in the testis. While it is conceivable that the testis can be infected by direct invasion caused by viremia, our results suggest that infected monocytes/macrophages migrating (e.g., from the lungs) may also be actively transporting and disseminating the virus into the testes [12]. Furthermore, we revealed a high SARS-CoV-2 infection and replication (according to morphological features in the cell cytoplasm) [13] in spermatogonia and macrophages, which are known to express ACE2 and TMPRSS2 [6, 14].

Previous research indicated that SARS-CoV-2 negativity in RT-qPCR tests (at least in two consecutive assays) usually occurs between 6 and 12 days from the onset of symptoms [15]. However, we found that the virus remains infective after a long infection period in the testes. It is well known that the testicular immune privilege prevents the autoimmune attack of haploid germ cells but allows viruses to escape immunosurveillance [16]. Thus, our findings suggest that the testes maintain infective viruses for extended periods in non-vaccinated severe COVID-19 patients. Furthermore, the testis environment has been related to delayed viral clearance in men compared to women [17]. Regarding these gender differences, it has been shown that male gonads express genes that increase SARS-CoV-2 susceptibility and severity [18]. In this context, the testes should not be neglected in evaluating the patient’s clinical condition because they may be a potential site of viral replication and, consequently, a source of viral load. However, further research to clarify whether or not the testes may serve as a viral reservoir for SARS-CoV-2 should be thoroughly pursued due to its potential impact on public health. To this end, identifying SARS-CoV-2 access to the testes and its potential replication sites is relevant since testicular immune tolerance may hamper viral clearance from the human body, as observed for other viruses [16].

It is known that the interaction of SARS-CoV-2 and ACE2 promotes this enzyme’s internalization, resulting in elevated levels of angiotensin II in the affected tissues [19]. Although overstimulation of the ANG II/AGT1R pathway underlies the damages observed in several tissues of COVID-19 patients [20], the present study is the first to show a role for this pathway in the testes. This molecular pathway was evidenced by the diminished expression of ACE2 (mainly in the tubular compartment), increased labeling of AGT1R in testicular cells, and elevated levels of angiotensin II. The influx of immune cells could have been mediated by angiotensin II, a potent pro-inflammatory molecule [21]. Furthermore, it is known that MCP1 and COX2 are stimulated by angiotensin II [22, 23].

Interestingly, an increased number of tryptase and chymase-positive mast cells and activated macrophages in the testes are associated with testicular inflammation and infertility in men [24, 25]. These cells can compromise the immune-privileged testicular environment, secreting pro-inflammatory cytokines, and interfering with testicular homeostasis [26]. Thus, the observed tubular damages may have occurred because of the high levels of angiotensin II [27], activated mast cells [28], and activated macrophages [29]. Furthermore, proteases and cytokines released by activated mast cells and macrophages are also known to disrupt tight epithelial junctions and deregulate the human blood-testis barrier [30].

In the intertubular compartment, the high levels of angiotensin II, mast cells producing histamines, and pro-inflammatory cytokines produced by macrophages can inhibit steroidogenesis [31, 32]. In line with Yang et al. [33], we showed the reduction in Leydig cell numbers and revealed, for the first time, that the intratesticular testosterone levels are 30 times reduced in the testes of patients with severe COVID-19. The low intratesticular testosterone levels observed herein may disturb the immune-privileged milieu [34] and compromise spermatogenesis [35]. In addition, we showed testis parenchyma’s fibrosis and vascular alterations. It is known that angiotensin II, activated mast cells, and activated macrophages stimulate collagen synthesis by fibroblasts [36–38] and increase vascular permeability [39–41]. In light of our findings, it can be speculated that the mechanisms discussed above may also be taking place in the testes of the severely affected patients studied herein. Further studies (e.g., utilizing human cell lines or animal models) are necessary to expand the proposed mechanisms and evaluate the long-term effects on testis function.

In relation to the patient’s clinical history, we did not observe a significant correlation between fever and testes damage. Recent data indicate that fever is not correlated with alterations in sperm quality following SARS-CoV-2 infection. We also showed that the more extended severe condition in ICU, the lower the number of surviving germ cells in the testes. Notably, the chronology of testicular pathogeny was based on the presence of advanced germ cells in the testis parenchyma and the time of ICU admission. Although our histological and molecular alterations reinforced this conceptual classification, the data of all individuals were also presented in a single group. However, analyzing the data of other studies, we noted that the worse testicular phenotype also seemed to be associated with the period of infection [4, 5]. Recent clinical results have shown that gonadal status may serve as a biomarker to identify patients with worse clinical outcomes and that extended hospitalization is linked to lower levels of total and free testosterone [42].

It is essential to mention that this study showed several molecular fluctuations during SARS-CoV-2 testicular pathogenesis. We provided a general overview, comparing testes’ cellular and molecular aspects from controls and deceased COVID-19 patients. We should point out the relevant limitations of the current study. Not all COVID-19 patients studied presented the same comorbidities. We observe the necessity of investigating the relationship between the comorbidities (e.g., diabetes and obesity) and the maintenance of the testicular immune privilege state. Although all patients in the present study had children (except for patient #9), we have no previous fertility data, such as hormonal and semen analyses. Controls were not submitted to the medications used for COVID-19 patients. On the other hand, we harvested and processed the testicles as soon as the patients died, in contrast with many cadaver studies showing long organ collection delays, which may have compromised the precise histology, detection of virion particles, and perception of peptides, mRNA, and hormonal fluctuations.

Finally, although some of the observations and proposed mechanisms might, at first, paint a dire scenario for severe COVID-19 disease, it is worth noticing that the present study only included non-vaccinated patients who died from COVID-19. Recent data on semen from mildly infected patients, who recovered from COVID-19, revealed that they reestablished their sperm quality after 3 months of the infection [43]. Nevertheless, our study reveals concerning aspects regarding the consequences of SARS-CoV-2 invasion of testicles that warrant further investigation by the scientific community at large.

## Conclusions

Our multidisciplinary findings might contribute to a better understanding of SARS-CoV-2 tropism, biology, and its impact on testes and male fertility. This is the first study that shows (1) the high SARS-CoV-2 tropism to the testis; (2) one mode of SARS-CoV-2 entrance in testes; (3) SARS-CoV-2 preferred infection and replication in spermatogonia and macrophages; (4) that the virus remains infective after a long infection period; (5) high levels of angiotensin II and activated mast cells and macrophages are critical players in promoting all testicular alterations; (6) the more extended and severe the disease, the lower the number of surviving germ cells; (7) fluctuation in the expression of several essential testicular genes; (8) that the intratesticular testosterone levels are 30 times reduced in testes of severely affected COVID-19 patients; (9) the prevalent types of collagen present in SARS-CoV-2 mediated testicular fibrosis; (10) the fluctuation of vasoconstrictive peptides in testes of non-vaccinated COVID-19 critically ill patients.

## Methods

### COVID-19 patients

From January 26 to March 4, 2021, we enrolled 11 non-vaccinated male patients deceased from COVID-19 complications, confirmed by SARS-CoV-2 RT-qPCR performed during their hospital stay in the Mater Dei Hospital Intensive Care Unit, Belo Horizonte, Brazil. All 11 patients were admitted to the ICU due to severe pulmonary symptoms (Table 1). The standard treatment at ICU included antibiotics, antimycotics, sedatives, muscular relaxants, analgesics, antihypertensives, inotropes, vasopressors agents, invasive mechanical ventilation, and hemodialysis. The Research Ethics Committee of the Mater Dei Hospital and the National Research Ethics Committee (CONEP) approved this investigation under the number CAAE: 30999320.1.0000.5128.

Postmortem collection of both testicles was performed after a legally responsible family member signed an informed consent document.

Testicles were collected through an incision on the median raphe of the scrotum. Two authors (MHF and YLG) collected all testicles no later than 3 h after the patient’s death. To perform viral and testicular genetic studies, fragments of testicular parenchyma were sampled and immersed into RNAlater® solution (Sigma-Aldrich). To investigate the viral replicative activity and testosterone and angiotensin levels, testis fragments were also sampled and then snap-frozen in liquid nitrogen. The remaining testicular halves were immersed in different fixatives, such as paraformaldehyde 4%, Bouin, methacarn, and glutaraldehyde 4%. Testis fragments were embedded in methacrylate, Epon 812 resin, and Paraplast® for histological, transmission electron microscopy, and immunohistochemistry analyses.

### Control patients

The control group was composed of six patients who underwent orchiectomy due to prostate cancer suspicion. These patients did not go through any treatment at the time of orchiectomy. Moreover, they exhibited normal spermatogenesis in seminiferous tubules. The age and hormonal levels of these patients are presented in Additional file 2: Table S1. Testicular fragments were obtained after the study was approved by the Ethics Committee in Research of the Universidade Federal de Minas Gerais COEP/UFMG (COEP ETIC n°117/07). All patients signed the informed consent.

Testicular samples were placed in liquid nitrogen, embedded in methacrylate, Epon, paraplast®, and conserved in RNAlatter. These samples were used for TEM, histological, hormonal, and molecular analyses. The mean age of patients was 58 years old, ranging from 46 to 65 years old.

### Detection OF SARS-CoV-2 in testis tissue

#### Genetic assays

##### SARS-CoV-2 detection using standard RT-qPCR

RNA of samples was extracted according to the protocol specified by the extraction kit (QIAamp® Viral RNA Mini Kit). The samples were stored in an ultra-freezer at − 80 °C. The collected samples were tested for the presence of SARS-CoV-2 viral RNA by RT-qPCR with primers to amplify the envelope (E) gene and the human transcript of the gene for RNAseP (RNP) as the endogenous control [44]. Samples were considered positive with a cycle threshold (CT) ≤ 40.

##### RT-qPCR using specific viral primers

Testes samples were macerated and submitted to RNA extraction using the Viral RNA Kit (Zymo Research, USA), following the manufacturer’s protocol. A two-step RT-qPCR approach was performed to optimize the detection of the viral RNA in the tissue samples and to avoid the possible influence of host cellular RNA. The obtained RNA was first submitted to cDNA synthesis using the CDC’s SARS-CoV-2 specific reverse primer 2019-nCoV_N1-R (TCT GGT TAC TGC CAG TTG AAT CTG) and the SuperScript™ III First-Strand Synthesis System (Invitrogen, Brazil).

The viral cDNAs were then amplified in a qPCR reaction using the GoTaq qPCR Master Mix (Promega, USA). We used both N1 primers from CDC’s SARS-CoV-2 detection protocol (2019-nCoV_N1-F: GAC CCC AAA ATC AGC GAA AT; 2019-nCoV_N1-R: TCT GGT TAC TGC CAG TTG AAT CTG) and followed the cycling recommendation indicated by the enzyme’s supplier in a QuantStudio 3 Real-Time PCR System (Applied Biosystems, USA). To normalize the results, the same process was performed to amplify the human β-actin control.

### Protein assays

#### Nanosensor

Gold nanorods (GNRs) were synthesized by the seed-mediated growth method as previously described [45]. The nanoparticles contained an average aspect ratio of 10 × 38 nm and a light absorbance peak of 713 nm. They were covalently functionalized with the polyclonal antibody anti-Spike protein (Rhea Biotech, Brazil) and a polyclonal antibody anti-Nucleocapsid protein (CTVacinas, Brazil) through a carbodiimide-activated amidation reaction. The binding between the gold surface and the antibodies was mediated by adding a capping layer formed by α-lipoic acid. A 2 mM α-lipoic acid solution (LA; Sigma Aldrich, USA) in ethanol was added to the GNR suspension (0.039 mg/mL). These suspensions were exposed to an ultrasonic bath (UNIQUE model U5C1850, 154W, 25KHz) at 55 °C for 30 min. The suspension was sonicated again for 2 h at 30 °C and left to rest overnight at RT to stabilize the interaction. GNRs were then centrifuged at 5600 g for 10 min and suspended in an aqueous solution. The GNR-LA complexes were kept at 4 °C in the dark. Next, the modified GNRs were re-dispersed in a 10 mM phosphate buffer containing 16 mM EDAC and 4 mM sulfo-NHS (30 min in an ice-bath under sonication). After another centrifugation step, GNR-LA suspensions were blocked with poly (ethylene glycol)-thiolate (5kD mPEG-SH, 10-4 mM, from Nanocs) for 10 min in an ice bath, under stirring (Additional file 1: Fig. S1a-b).

TEM images of the nanosensors were obtained on a 120 kV FEI Technai G2-12 (Spirit BioTwin, USA) microscope (Additional file 1: Fig. S1c). Samples were directly dripped onto a holey carbon film supported on a copper grid (400 mesh) (Pelco®, USA) without any further processing. Zeta potential was obtained with a Zetasizer Nano ZS90 analyzer from Malvern at an angle of 173° at RT, as previously described [7, 46, 47]. The nanoparticle’s size and zeta potential were measured simultaneously three times and in triplicate (Additional file 1: Fig. S1d-e).

Samples’ labeling and measurements were previously described [7]. Briefly, GNR-S were incubated with 1mg/mL of anti-S protein polyclonal antibody (Rhea Biotech, Brazil). Next, the samples were labeled with goat anti-rabbit IgG-CFL 488, 100 μg/mL λex: 488 nm, λem: 520 nm (Santa Cruz Biotechnology, USA) (Additional file 1: Fig. S1f). For GNR-N, samples were incubated with 10 μg of a polyclonal antibody anti-protein N for 60 min under axial shaking. Unbound antibodies were blocked with BSA (1%) for 30 min. Next, samples were labeled with Alexa Fluor® 546 goat anti-rabbit IgG H & L, 5 μg/mL λex: 540 nm, λem: 585 nm (Invitrogen, USA) and incubated under continuous shaking in the dark and at RT for 30 min (Additional file 1: Fig. S1g). Samples were measured using the spectrophotometer Varioskan Flash spectral scanning multimode reader (Thermo Scientific, USA).

To determine the concentration of antibodies in the LSPR-nanosensor, an antibody curve was carried out ranging from 0.25 μg to 10 μg for anti-S and anti-N proteins, respectively (Additional file 1: Fig. S1h-l). The concentration chosen was 0.5 μg for both GNR-S and GNR-N nanosensors. Afterward, the nanosensors were exposed to purified S- and N-proteins, ranging from 10 μg to 10 fg, and their binding affinity was assessed by UV-Vis spectroscopy (Thermo Scientific, USA).

Finally, the GNR-S and GNR-N nanosensors were exposed to macerated testes samples (obtained from the 11 patients with COVID-19), diluted in PBS-1X, for 30 min at 4 °C under sonication, and their respective LSPR were measured using optical plates (Costar, USA), by UV-Vis spectroscopy (Thermo Scientific, USA). Spectra analyses were performed using OriginPro version 9.0. We normalized and smoothed the curves and measured the biosensing event through the *X*-axis intercept of the derivative of the Gaussian peak for each patient. We compared the COVID-19 patient curves to the GNR and negative control patient curves (Additional file 1: Fig. S1m-q). We focused on the redshift that occurred at point zero (derivative axis) of the longitudinal peak. In this analysis, 5nm or below shifts were not considered significant.

#### Immunofluorescence against Spike protein

Immunofluorescence was performed using a validated primary anti-S protein antibody [48] (Rhea Biotech, Brazil) to detect and corroborate the viral presence in the testicular parenchyma (Additional file 2: Table S2). Reactions were visualized using Alexa-488 (Thermo Fisher Scientific, USA) conjugated secondary antibody, and images were acquired using a Nikon Eclipse Ti fluorescence microscope. As controls, we performed three different assays: (1) we used the testes of controls (prostate cancer patients); (2) we omitted the primary antibody in the testes of COVID-19 patients; and (3) we performed a negative control antigen, incubating the primary antibody with purified Spike protein (1:10; donated by CT-Vacinas-UFMG) before the immunofluorescence assay (Additional file 1: Fig. S2m-p).

### SARS-CoV-2 activity and replication

#### VERO cell exposition to testicular macerates

In the BSL-3 laboratory, testis samples from the 11 COVID-19 patients were macerated using the tissue Lyser (Tissuelyser II Retsch) and metallic beads (Qiagen, USA). All fragments were disrupted in 600 μL of DMEM without serum, and 300 μL was used for infection in vitro. VERO CCL-81 cells were seeded in a 6-well plate (Sarstedt, Germany) under 90% confluence. Culture media was discarded, and testis macerates were placed in the cell culture system for 1 h, at 37 °C and 5% CO_2_, with periodic homogenization. We did a negative control, exposing VERO cells to DMEM, and a positive control, exposing VERO cells to DMEM with SARS-CoV-2 (Wuhan strain SARS-CoV-2; MOI 0.01; donated by Dr. Edison Luiz Durigon).

After incubation, 2 mL of fresh supplemented DMEM was added, and cells were maintained (at 37 °C and 5% CO_2_) for 48 h. Next, 300 μL of each sample were adsorbed in another 6-well plate containing Vero CCL-81 cells for 1 h, at 37°C and 5% CO_2_, with periodic homogenization. Afterward, 2 mL of fresh DMEM was added, and cells were incubated. In parallel, 200 μL of each supernatant was collected (1st and 2nd day; Table 2), inactivated in 200 μL of Isothiocyanate of Guanidine, and frozen at – 80 °C for the RT-qPCR analysis.

#### Viral detection in the supernatant

To confirm the isolation of SARS-CoV-2, we extracted the total RNA from the supernatant (200 μl) using a using PureLink RNA Mini Kit (Invitrogen, USA), and the RT-qPCR reaction was performed using iTaq Universal Probes One-Step Kit (Bio-Rad). The SARS-CoV nucleocapsid RNA (N1) was measured with the following primer-probe set: F 5′-GACCCCAAAATCAGCGAAAT-3′, R 5′-TCTGGTTACTGCCAGTTGAATCTG-3′, and Probe 5′-FAM-ACCCCGCAT/ZEN/TACGTTTGGTGGACC-3IABkFQ-3′. The human transcript of the gene for RNAseP (RNP) was used as the endogenous control.

#### SARS-CoV-2 genomic and subgenomic RNA evaluation in VERO cells

Cytopathic effects were monitored, and the results were compared with a Mock-infection and concentrated SARS-CoV-2 virus as a positive control. After two passages, infected and control Vero CCL-81 cells were detached with Trypsin (Gibco, USA), spun down, and the concentrated pellets were kept frozen at – 80 °C for the RT-qPCR analysis. After cell lysis using Tissuelyser II Retsch and metallic beads (Qiagen, USA), 200 μL of each sample was added to 200 μL of lysis buffer with 2-mercaptoethanol. After homogenization, 200 μL of 70% ethanol was added. After washing with buffers, we added 40 μL of RNase-free water, and the samples were centrifuged for 1min 12000 × g to collect the total RNA.

SARS-CoV-2 genomic RNA was measured with the following primer-probe set: CoV-F3 (5′-TCCTGGTGATTCTTCTTCAGGT-3′), CoV-R3 (5′-TCTGAGAGAGGGTCAAGTGC-3′) and CoV-P3 (5′-AGCTGCAGCACCAGCTGTCCA-3′). SARS-CoV-2 subgenomic RNA was measured with the following primer-probe set: CoV-sgRNA-F (5′-CGATCTCTTGTAGATCTGTTCTC-3′), CoV-sgRNA-R (5′-ATATTGCAGCAGTACGCACACA-3′), and CoV-sgRNA-P (5′-ACACTAGCCATCCTTACTGCGCTTCG-3′).

#### Immunofluorescence and immunoperoxidase in Vero cells

To confirm viral isolation from the patient’s testes, we performed an immunofluorescence assay using the anti-S protein commercial antibody (Abcam, USA). The cells were seeded in a 96-well Black plate with a transparent bottom (Costar, Corning Incorporated, USA), and 50 μL of each supernatant from the third passage were adsorbed for 1 h, at 37 °C and 5% CO_2_, with periodic homogenization. Next, 50 μL of supplemented DMEM was added to each well and kept incubated for 4 h.

After the VERO infection, the contents were removed, and cell fixation was carried out using Paraformaldehyde 4% (BioRad, USA) for 15 min. The plates were washed with PBS 1X and blocked with a PBS solution containing 5% of Donkey Serum for 1h. The cells were then incubated with the anti-S antibody (Abcam, USA) for 1 h at 37°C. Afterward, cells were rinsed with PBS 1X and incubated with secondary antibody Alexa Fluor 488 anti-rabbit (Invitrogen, USA) and DAPI 300nM (dilution 1:1000) for 2 h at dark and room temperature. Finally, cells were washed with PBS 1X, and images were taken using EVOS 5000 ML (Invitrogen, USA).

We performed an immunohistochemistry assay using the anti-S commercial antibody (Rhea Biotech, Brazil). After fixation, the plates were blocked with a PBS solution containing 3% FBS for 15 min. Then, the cells were incubated overnight with the anti-S antibody (dilution 1:500) at RT. The cells were also incubated with an anti-rabbit IgG antibody conjugated to horseradish peroxidase (Promega, USA), diluted at 1:2500 at RT for 60 min. Immunoassay was revealed using the KPL TrueBlue Peroxidase Substrate (SeraCare, USA) for 10 min at RT, under gentle stir.

### Histomorphometric analysis

All testicular slides were scanned using the Panoramic MIDI II slide scanner (3DHISTECH, Hungary). The histomorphometric analyses were performed using the CaseViewer software (3DHISTECH, Hungary) and the Image J v.1.45s software (Image Processing and Analysis, in Java, USA).

#### Seminiferous epithelium cell composition

To describe the seminiferous epithelium integrity, at least 50 seminiferous tubule cross-sections were evaluated and classified according to the germ cell composition in the seminiferous epithelium. According to this classification, the patients were then categorized into three phases, as follows: phase 1, cross-sections containing all germ cell layers (spermatogonia, spermatocytes, and spermatids); phase 2, cross-sections containing spermatogonia and spermatocytes; phase 3, cross-sections containing only spermatogonia and/or degenerating/Sertoli cell-only seminiferous tubules. The results are presented as the percentage of seminiferous tubules in each category. After this analysis, we examined the clinical data of COVID-19 patients to understand the evolution of testicular pathogeny.

#### Seminiferous tubule measurements

Seminiferous tubules were analyzed using computer-assisted image analysis of 30 randomly chosen seminiferous tubules cross-sections per donor. To determine the seminiferous tubule diameter and tunica propria width, the measurements were taken at × 400 magnification, and the results were expressed in micrometers.

#### Leydig cell volume density

The volume densities (%) of testicular tissue components were obtained after counting 7200 points over testis parenchyma. The intersections that coincided with Leydig cells were counted in 15 randomly chosen fields by horizontal scanning of the histological sections at × 200 magnification [49].

#### Hemorrhagic scores

As red cell bleeding was a common finding, this pathology was measured in four scores, as follows:

1. Patients who presented many red blood cells inside the seminiferous tubule lumen and intertubular compartment (score 3);
2. Patients with vast areas of red blood cells bleeding in the intertubular compartment (score 2);
3. Patients with small areas of red blood cell bleeding in the intertubular compartment (score 1);
4. Patients without red blood cell bleeding (score 0).

### Histochemistry techniques

#### Mast cell counts

Toluidine-blue staining was used to determine the number of mast cells. We investigated 15 testicular fields (× 20 magnification), and the cells were quantified per patient.

#### PAS staining

A Schiffs kit (Sigma-Aldrich, USA) was used for PAS staining, as per the manufacturer’s protocol. In brief, the sections were pre-treated with periodic acid for 5 min at RT, slowly rinsed in distilled water, and then stained with Schiffs solution for 10 min at RT in the dark. The nuclei were stained with hematoxylin for 5 min at RT, followed by six dips in 1% hydrochloric alcohol. After dehydration with 70, 90, and 100% graded alcohol, the sections were immersed twice in xylene for 10 min each. Then, the slides were mounted with a coverslip and sealed with Entellan resin (Sigma-Aldrich, USA). Images were captured using light Olympus microscopy (BX-60).

#### Masson’s Trichrome and Picrosirius red

Tissue samples were stained with Masson’s Trichrome and Picrosirius red to assess fibrosis and collagen types I and III. Images of the two techniques were captured in a Spot Insight Color digital camera adapted for Olympus BX-40, using the Spot software version 3.4.5. To determine the area of fibrosis and differentiate the types of collagens, images of three random areas of each patient were obtained at × 100 magnification. Images were analyzed with Image J v. 1.53c software (National Institutes of Health, USA) using the Color Deconvolution tool, getting the average of the three areas evaluated [50].

### Immunostaining

For immunostaining, deparaffinized sections (5 μm thick) were dehydrated and submitted to heat-induced antigenic recovery (water bath) with buffered sodium citrate (pH 6.0) at 90 °C for 40 min. Then, the sections were immersed in BSA 3% (in PBS) solution to block non-specific antibody binding and incubated overnight at 4 °C with primary antibodies (Additional file 2: Table S2). Reactions were visualized using biotin-conjugated secondary antibodies (anti-goat: 1:100 dilution, Abcam, ab6740; anti-mouse: 1:200 dilution, Imuny, IC1M02; anti-rabbit: 1:200 dilution, Abcam, ab6720) combined with Elite ABC Kit (Vector Laboratories, USA). Signal detection was obtained via peroxidase substrate 3,39-diaminobenzidine (DAB; Sigma Aldrich, USA) reaction and counterstaining with Mayer’s hematoxylin (Merck, USA).

To identify targets of SARS-CoV-2 in the testis intertubular compartment, double-immunofluorescence anti-S with anti-CD-68 or anti-Chymase was performed. Reactions were visualized using Alexa-488 (1:100 dilution), Alexa-546 (1:200 dilution), and Alexa-633 (1:200 dilution), all from Thermo Fisher Scientific (USA), and conjugated secondary antibody. For ACE2 evaluation, we used anti-ACE2 (Proteintech, USA) and Alexa-594 (1:100 dilution) from Jackson Immunoresearch (USA). Images were acquired using the Nikon Eclipse Ti fluorescence microscope.

To analyze immunopositive macrophages and T lymphocytes, CD68 and CD3 positive cells were quantified by counting 10 testicular fields at × 400 magnification. Only cells with visible nuclei, brown cytoplasm, and morphology compatible with the evaluated cells were counted. Microvessel density analysis was also performed in 10 fields at × 400 magnification, and only CD31 immunopositive structures with or without lumen were counted (vessels containing muscle walls were not counted).

### Transmission electron microscopy

Testes fragments were fixed by immersion in 4% glutaraldehyde (EMS, USA). Smaller pieces (1–2 mm thickness) were obtained and postfixed in reduced osmium (1% OsO4 and 1.5% potassium ferrocyanide in distilled water) for 90 min, dehydrated in ethanol, and embedded in Araldite epoxy resin. Ultrathin sections (60 nm thick) were obtained using a diamond knife on a Leica EM UC6 ultramicrotome (Leica Microsystems) and mounted on 200 mesh copper grids (Ted Pella). The ultrathin sections were stained with lead citrate (Merck, USA) and analyzed using a transmission electron microscope (Tecnai G2-12 Thermo Fisher Scientific/FEI, USA). The viral particle was defined when presenting a round to oval shape, a membrane, an electron-dense interior, and within a membrane compartment [51].

### Enzymatic and hormone measurements

The enzymatic activity of MPO and NAG and the total concentration of testosterone and angiotensin II were determined in human testis homogenates. For this purpose, 50 mg of snap-frozen testis from deceased COVID-19 patients and controls were homogenized in 450 μL cold PBS supplemented with a protease inhibitor cocktail (Cat n° S8830, Sigma-Aldrich). After three freeze/thaw cycles in a liquid nitrogen/water bath (37 °C), the samples were centrifugated (14.000 g, 10 min, 4 °C), and the supernatants were collected.

For MPO and NAG measurements, 100 μL of tissue homogenates were mixed 1:1 in MPO buffer assay (0,1M Na3PO4, 0.1% [w/v] HETAB, pH 5.0) or NAG buffer assay (0.2M citric acid, 0.2M Na2HPO4, pH 4.5), respectively, just prior the freeze/thaw step. The activity of MPO and NAG, which is an indirect estimation of the abundance of neutrophils and macrophages, was measured in a colorimetric enzymatic assay. Intratesticular testosterone levels were determined in testis homogenates using a chemiluminometric immunoassay run on the Atellica IM Analyzer (Siemens Healthcare Diagnostics). The concentration of Angiotensin II was measured by ELISA, according to the procedures supplied by the manufacturer (MyBioSource, San Diego, CA, USA). The kit applied the sandwich ELISA technique. The sensitivity of the assay was 12 pg/mL.

### Testicular gene expressions

Total RNA was isolated from testes using AurumTM Total RNA MiniKit® (BioRad, USA). A Nanodrop spectrophotometer (Thermo Fischer, USA) was used to measure the quantity and integrity of total RNA. RNA (2 μg/sample) was reverse transcribed using the iScript cDNA Synthesis Kit (BioRad, USA). cDNAs (10 ng) were amplified by qPCR with iTaq Universal SYBR Green Supermix (BioRad, USA) in Rotor-Gene Q (Qiagen, USA). The primer sequences used can be found in Additional file 2: Table S2. Relative levels of expression were determined by normalization to RPL19 e HPRT1 using the ∆∆CT method. The testicular gene expressions were displayed as Heat maps (Fig. 5 and Fig. 6) and individual graphs (Additional file 1: Fig. S6 and Additional file 1: Fig. S7).

### Statistical analyses

Demographics and clinical characteristics were presented using descriptive statistics: mean and standard deviation (SD) for normally distributed continuous data, median and interquartile range (IQR) for non-normally distributed continuous data, and proportions and frequencies for categorical data. The presence and the strength of a linear relationship between demographics and clinical characteristics and the damage to the testicles were analyzed using Spearman correlation.

All quantitative data were tested for normality and homoscedasticity of the variances following Kolmogorov–Smirnov (Dallal–Wilkinson–Lilliefor) and Bartlett tests. Data from the fluorometry assay analyses were evaluated by one-way ANOVA for comparisons within groups, followed by Newman-Keuls (normal distribution). Histomorphometric and gene expression data were analyzed by unpaired Student’s *t*-test, comparing COVID-19 groups to controls and COVID-19-P1 to COVID-19-P2 (COVID-19-P3 was not considered for statistics). The data obtained were represented as the mean ± SEM and geometric mean ± SD. Graphs and statistical analyses were conducted using GraphPad PRISM v6.0 (GraphPad Software, Inc.). Differences were considered statistically significant at *p* < 0.05.The individual raw data are available online [52]. 

## Supplementary Information

**Additional file 1: Fig. S1.** COVID-19 nanosensor platforms. **Fig. S2.** Immunolabeling against S-protein in testes of all COVID-19 patients and in VERO cells. **Fig. S3.** Histology of activated mast cells and infiltrative monocytes in COVID-19 patients. **Fig. S4.** Tubular compartment morphological alterations. **Fig. S5.** Leydig cell, collagen deposition, and blood vessel alterations in COVID-19 patients. **Fig. S6.** Transcript level of key genes related to high angiotensin II levels, immune cells and vascular system and fibrosis. **Fig. S7.** Relative expression of important genes associated with the tubular compartment and Leydig cells. Individual values can be found at https://doi.org/10.6084/m9.figshare.16777786.v8.

**Additional file 2: Table S1.** Clinical data from control patients. **Table S2.** Antibodies and Primers (qPCR) used in this study.

## Data Availability

All data generated or analyzed during this study are included in this published article and its supplementary information files. The datasets generated and/or analyzed during the current study are also available in figshare. 10.6084/m9.figshare.16777786.v8

## Abbreviations

17BHSD: 17 beta-hydroxysteroid dehydrogenase
3BHSD: 3 beta-hydroxysteroid dehydrogenase
ACE2: Angiotensin-converting enzyme 2
AGT1R: Angiotensin II receptor type 1
ANG II: Angiotensin II
BAD: BCL2 associated agonist of cell death
BAX: BCL2 associated X protein
BSL-3: Biosafety level 3
CMA1: Chymase 1
Col3a: Collagen type III alpha 1 chain
COVID-19: Coronavirus disease 2019
COX2: Cyclooxygenase-2
CT: Cycle threshold
DAZL: Deleted in azoospermia like
DMV: Double-membrane vesicles
E gene: Envelope gene
ERGIC: Endoplasmic reticulum Golgi intermediate complex
GNR: Gold nanorods
HPRT1: Hypoxanthine phosphoribosyltransferase 1
HRH4: Histamine H4 receptor
ICU: Intensive care unit
LHR: Luteinizing hormone receptor
LSPR: Localized surface plasmon resonance
MCP1: Monocyte chemoattractant protein-1
MPO: Myeloperoxidase
NAG: N-acetyl-BD-glucosaminidase
N-Protein: Nucleocapsid protein
PAS: Periodic acid–Schiff
PDGFA: Platelet derived growth factor subunit A
RMW: Replication membranous webs
RNP: Ribonuclease P/ribonucleoprotein
RPL19: Ribosomal protein L19
S-protein: Spike protein
STAR: Steroidogenic acute regulatory protein
TEM: Transmission electron microscopy
TMPRSS2: Transmembrane serine protease 2
TPSB2: Tryptase beta 2
VEGF: Vascular endothelial growth factor
