# Supplementary material for: High SARS-CoV-2 tropism and activation of immune cells in the testes of non-vaccinated deceased COVID-19 patients
Source: BMC Biol. 2023 Feb 16;21:36. doi: 10.1186/s12915-022-01497-8 (PMC9933832; doi:10.1186/s12915-022-01497-8)
Supplement: Supplementary file 1 — Additional file 1: Fig. S1. COVID-19 nanosensor platforms. Fig. S2. Immunolabeling against S-protein in testes of all COVID-19 patients and in VERO cells. Fig. S3. Histology of activated mast cells and infiltrative monocytes in COVID-19 patients. Fig. S4. Tubular compartment morphological alterations. Fig. S5. Leydig cell, collagen deposition, and blood vessel alterations in COVID-19 patients. Fig. S6. Transcript level of key genes related to high angiotensin II levels, immune cells and vascular system and fibrosis. Fig. S7. Relative expression of important genes associated with the tubular compartment and Leydig cells. Individual values can be found at https://doi.org/10.6084/m9.figshare.16777786.v8. [file 12915_2022_1497_MOESM1_ESM.pdf]

Fig. S1

Recognition of S and N proteins

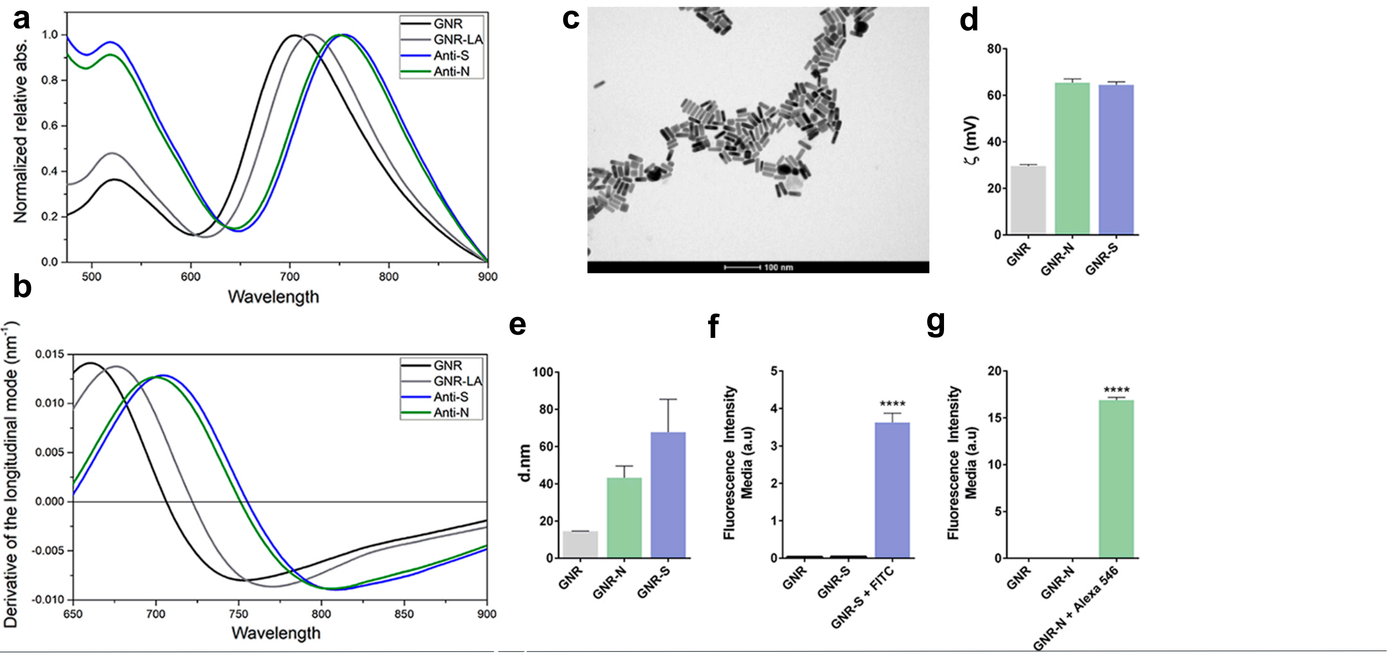

Limit of detection

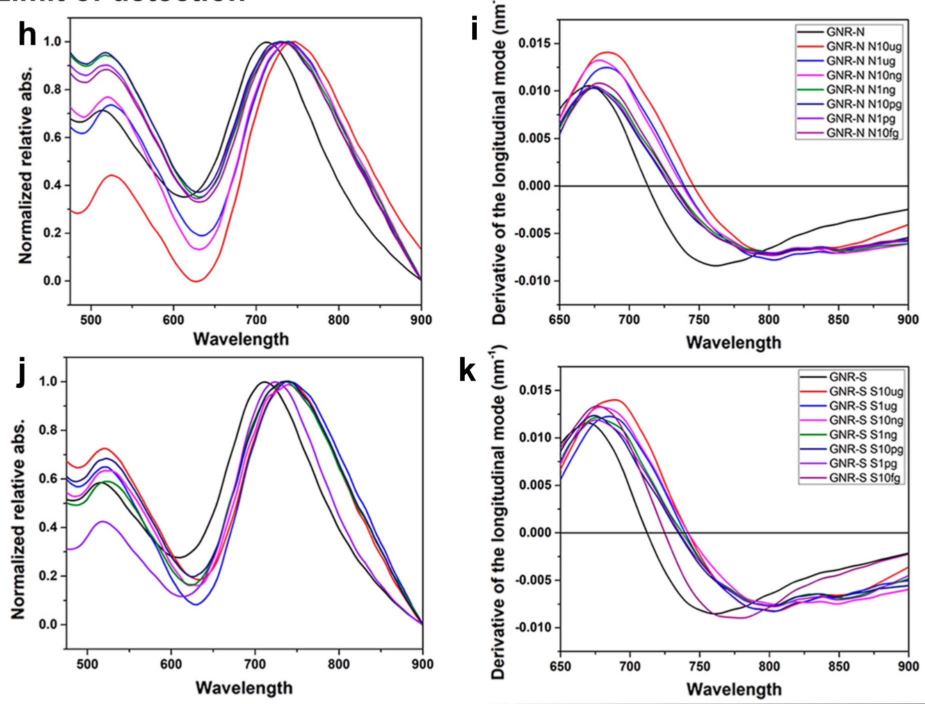

l

[ ]	Protein N (nm)	Protein S (nm)
10ug	33.4	29.5
1ug	27.6	27.3
10ng	24.7	29.5
1ng	19.6	24.4
10pg	16.9	22.2
1pg	17.5	21.5
10fg	17.6	12.2

Nano-sensor in testes of COVID-19 patients

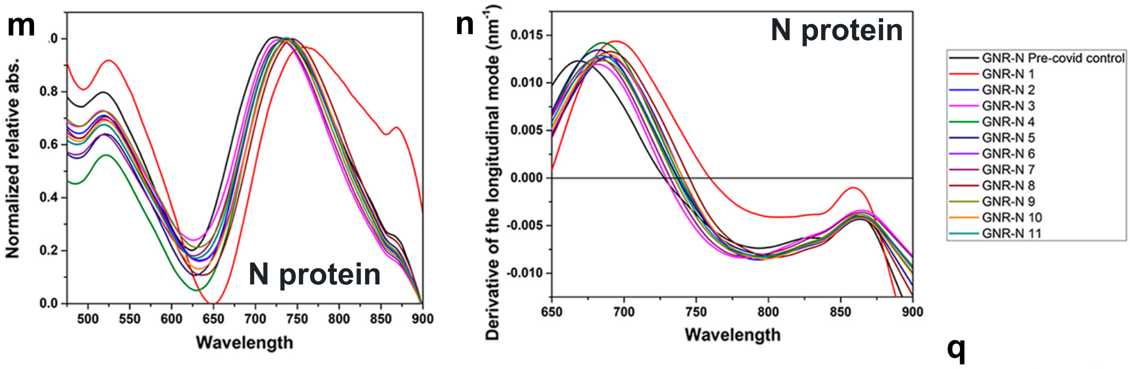

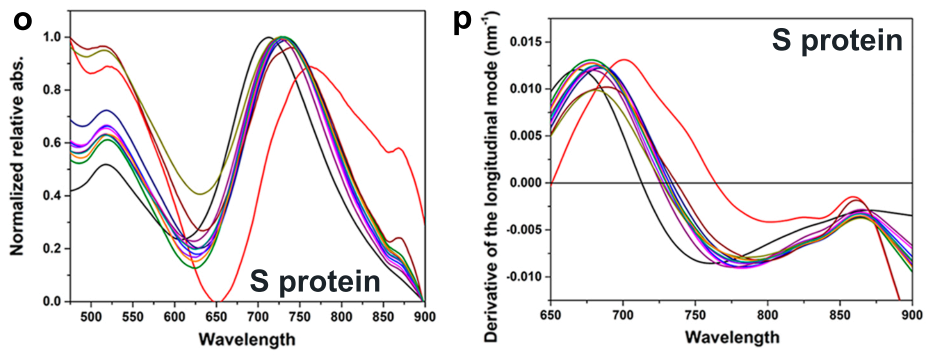

q

Sample	N shift (nm)	S shift (nm)
1	31.5	51
2	13.2	18.9
3	1.2	17.5
4	11.2	17
5	9.9	20.9
6	13.2	15
7	4.5	12.4
8	17.5	17
9	12.9	17.5
10	7.2	13.6
11	8.7	16.5

Fig. S1. COVID-19 nanosensor platforms. Gold nanorods (GNRs) were coated with anti-S and anti-N antibodies to construct a COVID-19 nanosensor and then characterized by different techniques. a) UV-Vis LSPR spectra of bare GNRs, lipoic-acid coated surface (GNR-LA) and GNR nanosensors recognizing S- and N-proteins, respectively. b) derivative curve of the observed light absorbance redshifts. c) TEM image of the GNRs. d) stability of both nanosensors by zeta potential measurements. e) the dynamic light scattering of both nanosensors and their respective hydrodynamic radius. f-g) fluorophores binding both nanosensors. h-k) Light absorbance spectra due to LSPR and respective derivative curves regarding the limits of detection of both nanosensors exposed to different concentrations of S- and N-proteins in means of nm shifting. m-p) Light absorbance spectra due to LSPR and respective derivative curves of the nanosensors recognizing the presence of SARS-CoV-2 in each sample compared to non-infected Control. q) the LSPR shift (nm) of each nanosensor after sample exposure. (mean ± SD, ****$p<0.001$)

Fig. S2

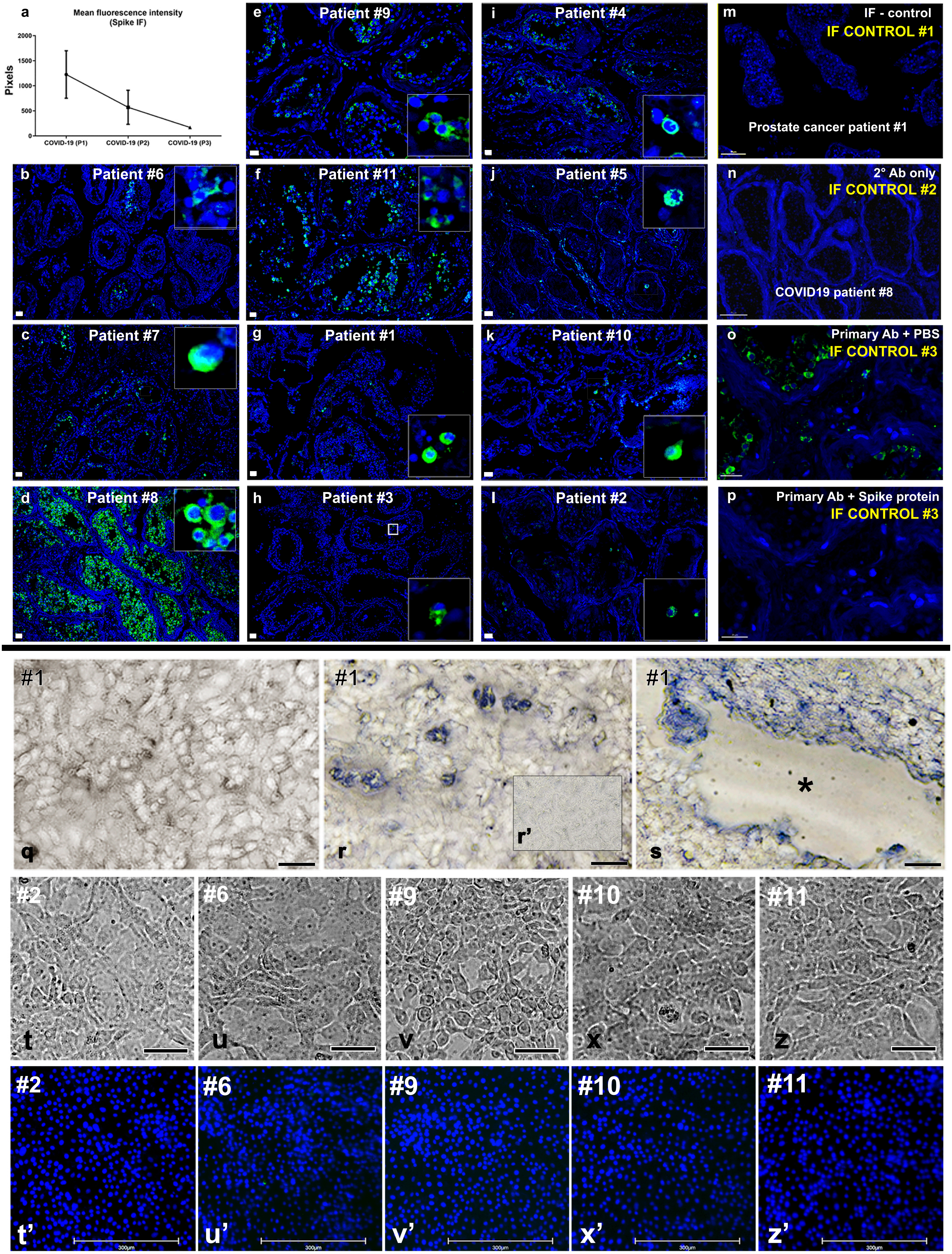
a
Mean fluorescence intensity
(Spike IF)
Pixels
2000
1500
1000
500
0
COVID-19 (P1)
COVID-19 (P2)
COVID-19 (P3)
b
Patient #6
c
Patient #7
d
Patient #8
e
Patient #9
f
Patient #11
g
Patient #1
h
Patient #3
i
Patient #4
j
Patient #5
k
Patient #10
l
Patient #2
m
IF - control
IF CONTROL #1
Prostate cancer patient #1
n
2° Ab only
IF CONTROL #2
COVID19 patient #8
o
Primary Ab + PBS
IF CONTROL #3
p
Primary Ab + Spike protein
IF CONTROL #3
#1
q
#1
r
r'
#1
s
*
#2
t
#6
u
#9
v
#10
x
#11
z
#2
t'
#6
u'
#9
v'
#10
x'
#11
z'

Fig. S2. Immunolabeling against S-protein in testes of all COVID-19 patients and in VERO cells. a) mean fluorescence index (in pixels) evaluating the large images of immunostainings against S-protein. b-l) images of testis parenchyma of COVID-19 patients. b-f) first phase COVID-19 patients. g-k) second phase COVID-19 patients. l) third phase COVID-19 patient. Inserts depict labeled germ cells in all patients. Scale bars = 20 µm. m-o) Controls of the immunofluorescence reactions. m) negative image of Control patient #1 (Scale bar = 70 µm). n) negative control, omitting the primary antibody. o-p) antigen control, comparing the tissue incubated with primary antibody (o) and primary antibody previously incubated with purified Spike protein. q-z') immunoperoxidase and immunofluorescence in VERO cells exposed to testicular macerates. q-s) VERO cells exposed to testicular macerate from patient #1. q) negative control. r) immunostaining (blue labeling) depicting S-protein in VERO cells. r') Negative control, omitting the primary antibody. s) image illustrating the cytopathic effect (*) in VERO cells. t-z) reduced cytopathic effect in VERO cells exposed to testicular macerates from patients #2, #6, #9, #10, and #11. Scale bars= 30µm. t'-z') no labeling for S-protein in VERO cells exposed to testicular macerates from patients #2, #6, #9, #10, and #11. Scale bars=300µm. Individual values can be found at: https://doi.org/10.6084/m9.figshare.16777786.v8.

Fig. S3

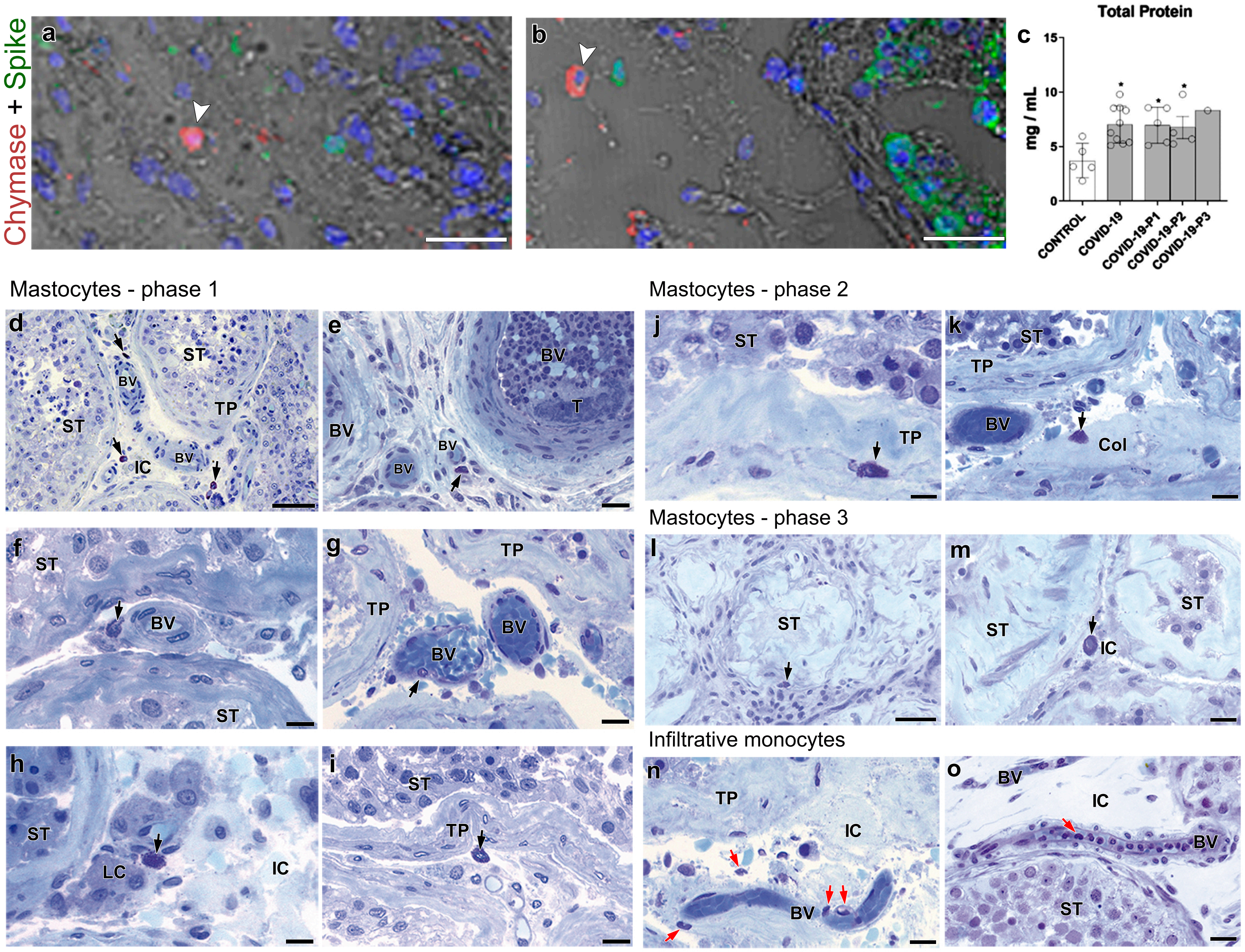
Chymase + Spike
a
b
c
Total Protein
mg / mL
15
10
5
0
CONTROL
COVID-19
COVID-19-P1
COVID-19-P2
COVID-19-P3
Mastocytes - phase 1
d
ST
BV
TP
ST
IC
BV
e
BV
T
BV
BV
BV
f
ST
BV
ST
g
TP
TP
BV
BV
h
ST
LC
IC
i
ST
TP
Mastocytes - phase 2
j
ST
TP
k
ST
TP
BV
Col
Mastocytes - phase 3
l
ST
m
ST
ST
IC
Infiltrative monocytes
n
TP
IC
BV
o
BV
IC
BV
ST

Fig. S3. Histology of activated mast cells and infiltrative monocytes in COVID-19 patients. a-b) activated mast cells (chymase +, white arrowheads) are not labeled for the S-protein (green). Blue = dapi staining. (Scale bars = 50 µm). c) quantity of protein per milligram of tissue indicating an inflammatory process (t-test; two-tailed; infected groups vs control, *$p<0.05$). d-m) a high number of mast cells in the testes of COVID-19 patients (arrows) (Scale bar = 50 µm). d-e) mast cells near blood vessels (BV) filled with immune cells (T represents a thrombotic area) (Scale bar = 20 µm). f-g) perivascular mast cells (arrows) near intact (f) and disrupted (g) blood vessels (Scale bars = 15 µm). h) mast cell (arrow) near Leydig cells (Scale bar = 15 µm). i) mast cell (arrow) near thickened tunica propria (Scale bar = 15 µm). j) mast cell (arrow) inside the seminiferous tubule (Scale bar = 15 µm). k) mast cell (arrow) near a collagen matrix (Col) in the intertubular space (Scale bar = 15 µm). l) mast cell (arrow) inside a fibrotic seminiferous tubule (Scale bar = 50 µm). m) mast cell in the intertubular area of a third-phase patient (Scale bar = 25 µm). n-o) a high number of infiltrative monocytes (red arrows) in the intertubular compartment (IC) (Scale bars = 20 µm). Individual values can be found at: https://doi.org/10.6084/m9.figshare.16777786.v8.

Fig. S4

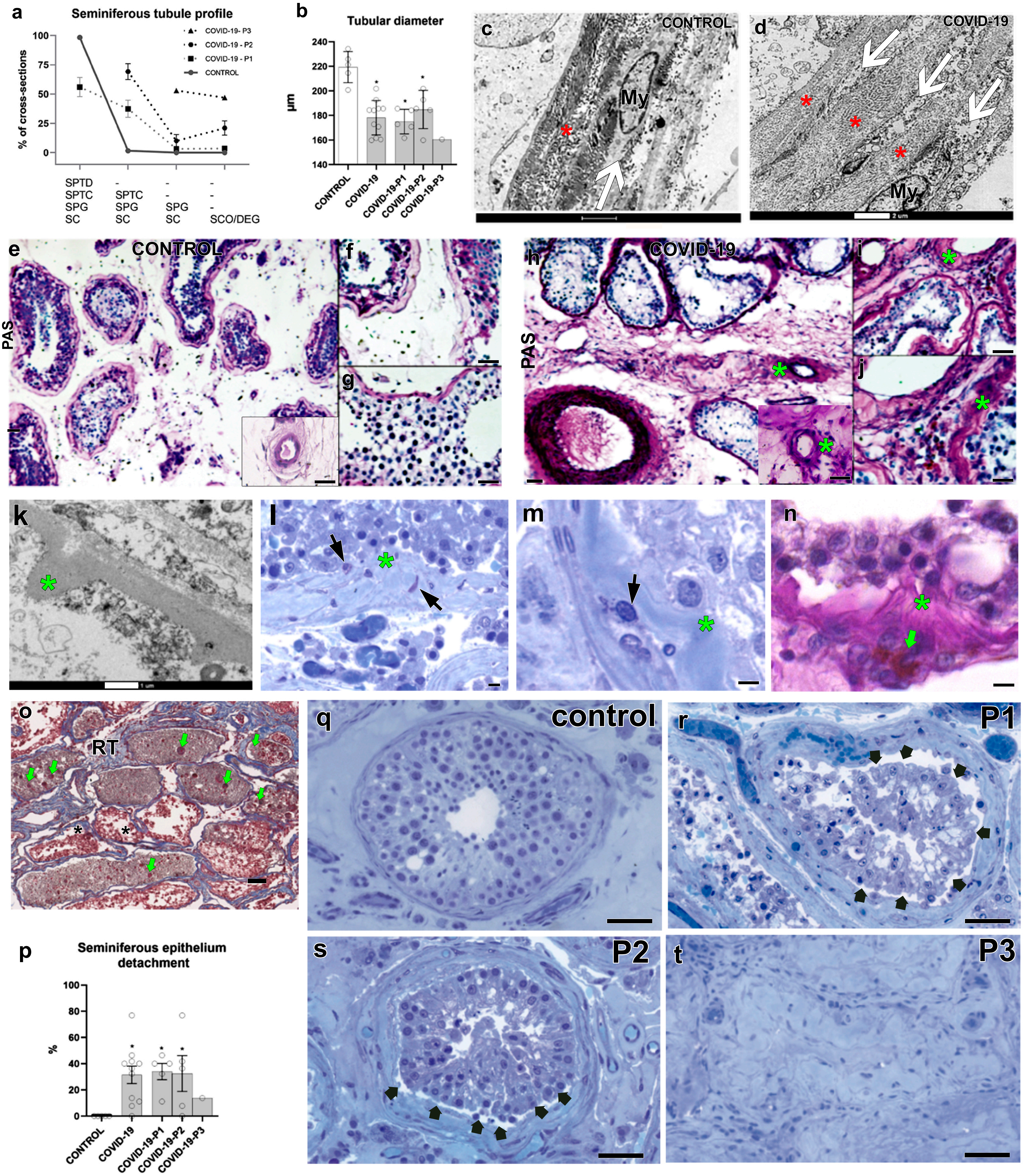
a
Seminiferous tubule profile
% of cross-sections
COVID-19- P3
COVID-19 - P2
COVID-19 - P1
CONTROL
SPTD
SPTC
SPG
SC
SCO/DEG
b
Tubular diameter
µm
CONTROL
COVID-19
COVID-19-P1
COVID-19-P2
COVID-19-P3
c
CONTROL
My
d
COVID-19
My
e
CONTROL
PAS
f
g
h
COVID-19
PAS
i
j
k
l
m
n
o
RT
p
Seminiferous epithelium detachment
%
q
control
r
P1
s
P2
t
P3

Fig. S4. Tubular compartment morphological alterations. a) quantification of the seminiferous tubule cross-sections profile (in %) in Controls and COVID-19 patients. b) seminiferous tubule diameter in Controls and COVID-19 patients (t-test; two-tailed; infected groups vs control, *$p<0.05$). c-d) a high number of collagen fibers (asterisks) and peritubular myoid cells (My, white arrows) in COVID-19 patients (Scale bars = 2 µm). e-j) basement membrane (PAS+) in Controls (e-g) and COVID-19 patients (h-j) (Scale bars = c: 30 µm). Asterisks denote high deposition of glycoproteins in the basement membrane and surrounding blood vessels. k-n) convoluted appearance of the basement membrane of COVID-19 patients (asterisks) (Scale bars = s: 1 µm; t-v: 10 µm). Mast cells (black arrows) and macrophages (green arrows) were observed near these areas. o) rete testis (RT) area filled with sloughing and apoptotic germ cells (green arrows) in COVID-19 patients (Scale bar = 50 µm). p-t) seminiferous epithelium detachment (black arrows) from the tunica propria in Controls and COVID-19 patients (t-test; two-tailed; infected groups vs control, *$p<0.05$) (Scale bars = 50 µm). Individual values can be found at: https://doi.org/10.6084/m9.figshare.16777786.v8.

FigS5

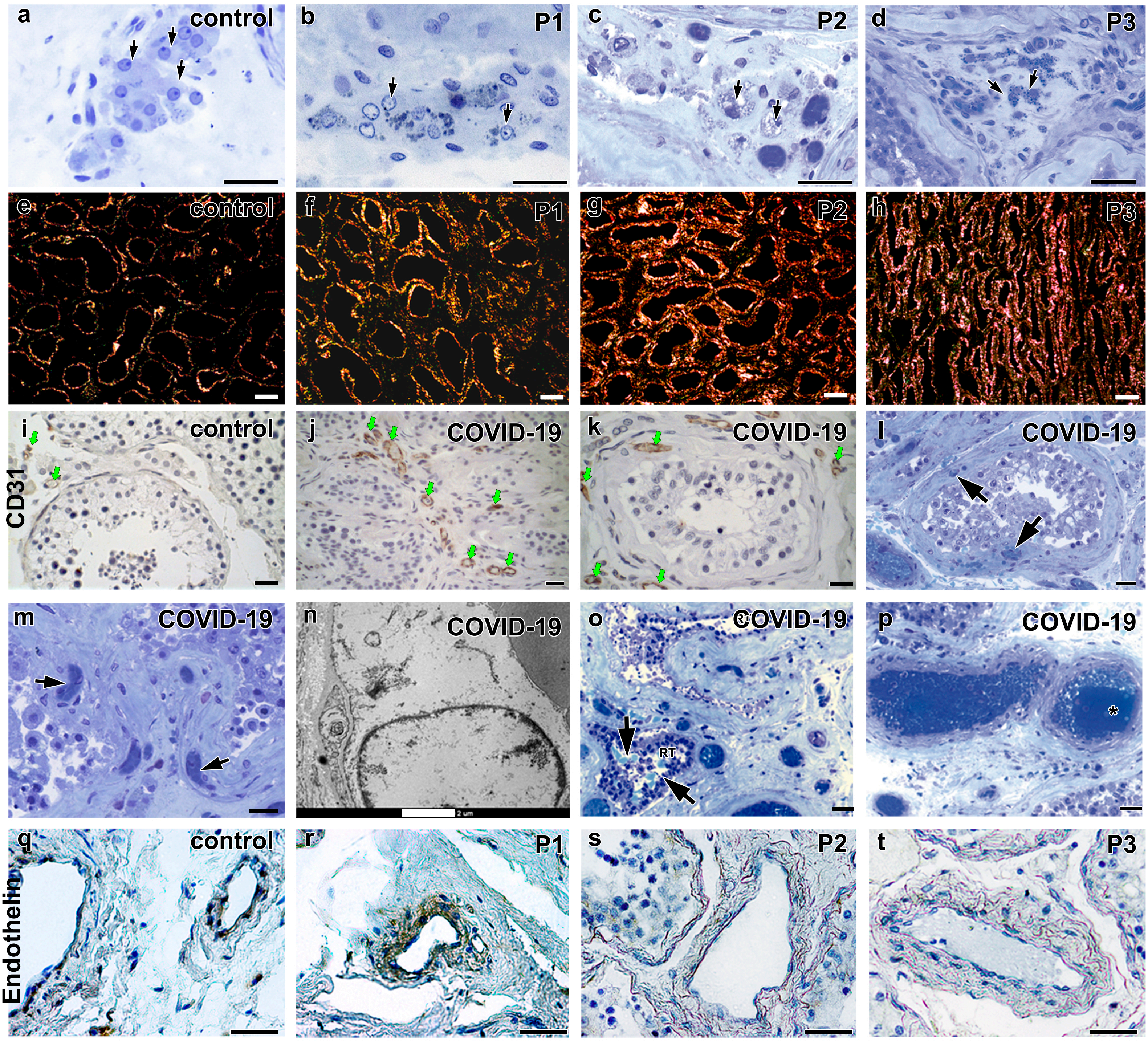
a
control
b
P1
c
P2
d
P3
e
control
f
P1
g
P2
h
P3
CD31
i
control
j
COVID-19
k
COVID-19
l
COVID-19
m
COVID-19
n
COVID-19
2 um
o
COVID-19
RT
p
COVID-19
*
Endothelin
q
control
r
P1
s
P2
t
P3

Fig. S5. Leydig cell, collagen deposition, and blood vessel alterations in COVID-19 patients. a-d) Leydig cell morphology in Controls and COVID-19 patients (Scale bars = 30 µm). b-d) vacuoles and granules in the Leydig cell cytoplasm. e-h) Picrossirius staining demonstrating Birefringence in Yellow Orange (type I collagen) and green (type III collagen) (Scale bars = 100 µm). i-k) CD31 immunolabeling in Controls (i) and COVID-19 patients (j-k) evidencing the newly formed blood vessels in testis parenchyma (Scale bar = 30 µm). l-m) blood vessels inside tunica propria (arrows) of COVID-19 patients (Scale bars = 30 µm). n) TEM image of an immature endothelial cell identified in tunica propria (Scale bar = 2 µm). o) red blood cells (arrows) inside the rete testis (RT) lumen (Scale bar = 30µm). p) thrombus (*) inside the blood vessel (Scale bar = 30 µm). q-t) Endothelin1/2/3 immunolabeling in Controls (q) and COVID-19 phase one, phase two, and phase three (p1, p2, and p3) patients showing a higher intensity in phase one patients followed by weak labeling in phases two and three patients (Scale bars= 50 µm).

Fig. S6

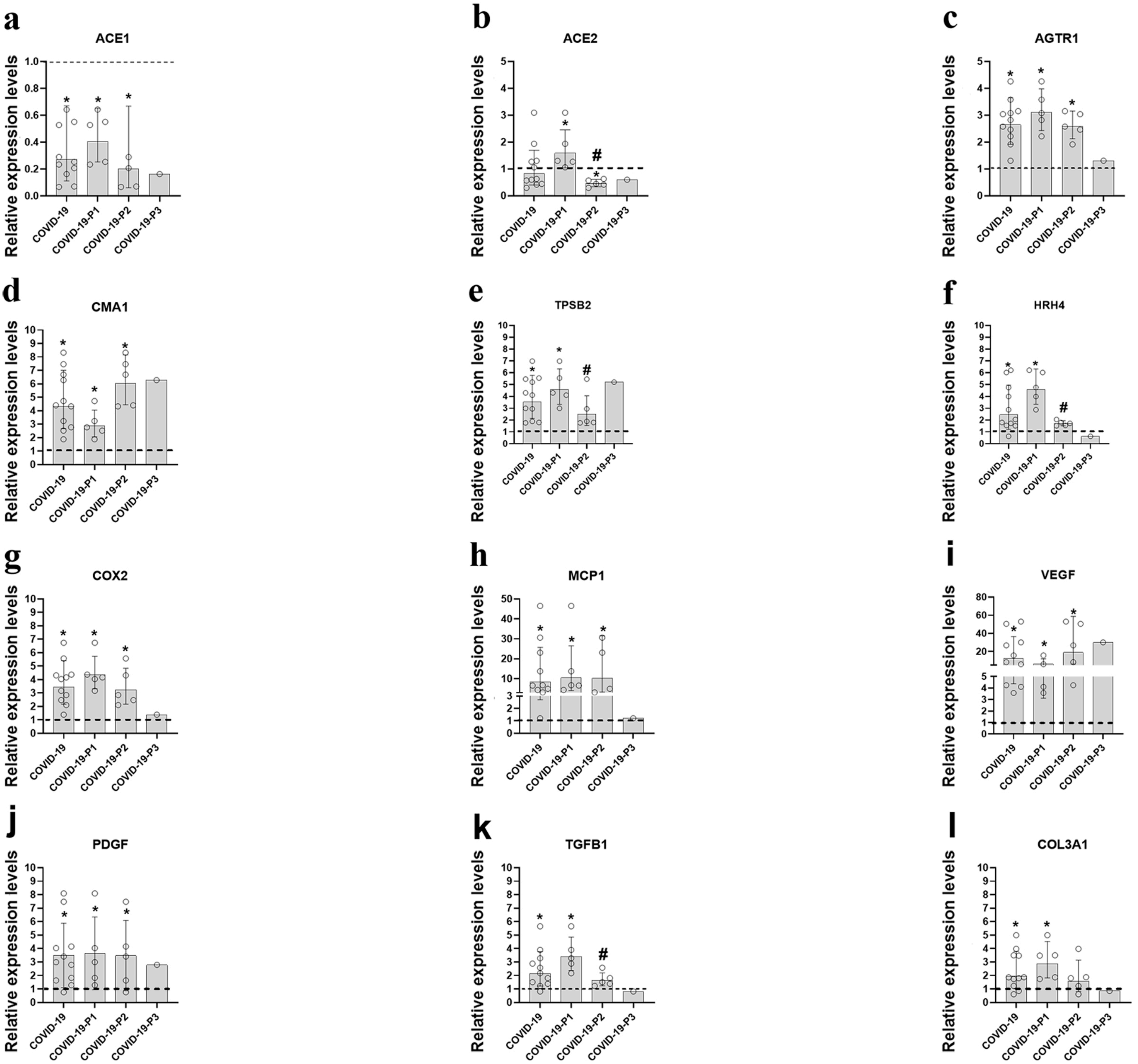
a
ACE1
b
ACE2
c
AGTR1
d
CMA1
e
TPSB2
f
HRH4
g
COX2
h
MCP1
i
VEGF
j
PDGF
k
TGFB1
l
COL3A1
Relative expression levels
COVID-19
COVID-19-P1
COVID-19-P2
COVID-19-P3

Fig. S6. Transcript level of key genes related to high angiotensin II levels, immune cells and vascular system and fibrosis. a-c) Genes from the renin-angiotensin system: ACE1 (a), ACE2 (b), and AGT1R (c). d-e) mast cell genes: chymase (CMA1) (d), and tryptase (TPSB2) (e). f) Histamine receptor (HRH4). g) Cyclooxygenase-2 (COX-2). h) Monocyte chemoattractant protein 1 (MCP1). i) Vascular Endothelial Growth Factor (VEGF). j) Platelet-Derived Growth Factor (PDGF). k) Transforming Growth Factor Beta 1 (TGFB1). l) Collagen Type III Alpha 1 Chain (COL3A1). Data are expressed as geometric mean ± SD. *$p < 0.05$ COVID-19 patients compared to the Control group (which is set at 1; dashed line). # indicates significant differences between COVID-19-P1 and COVID-19-P2 ($p < 0.05$); t-test (two-sided). Individual values can be found at: https://doi.org/10.6084/m9.figshare.16777786.v8.

Fig. S7

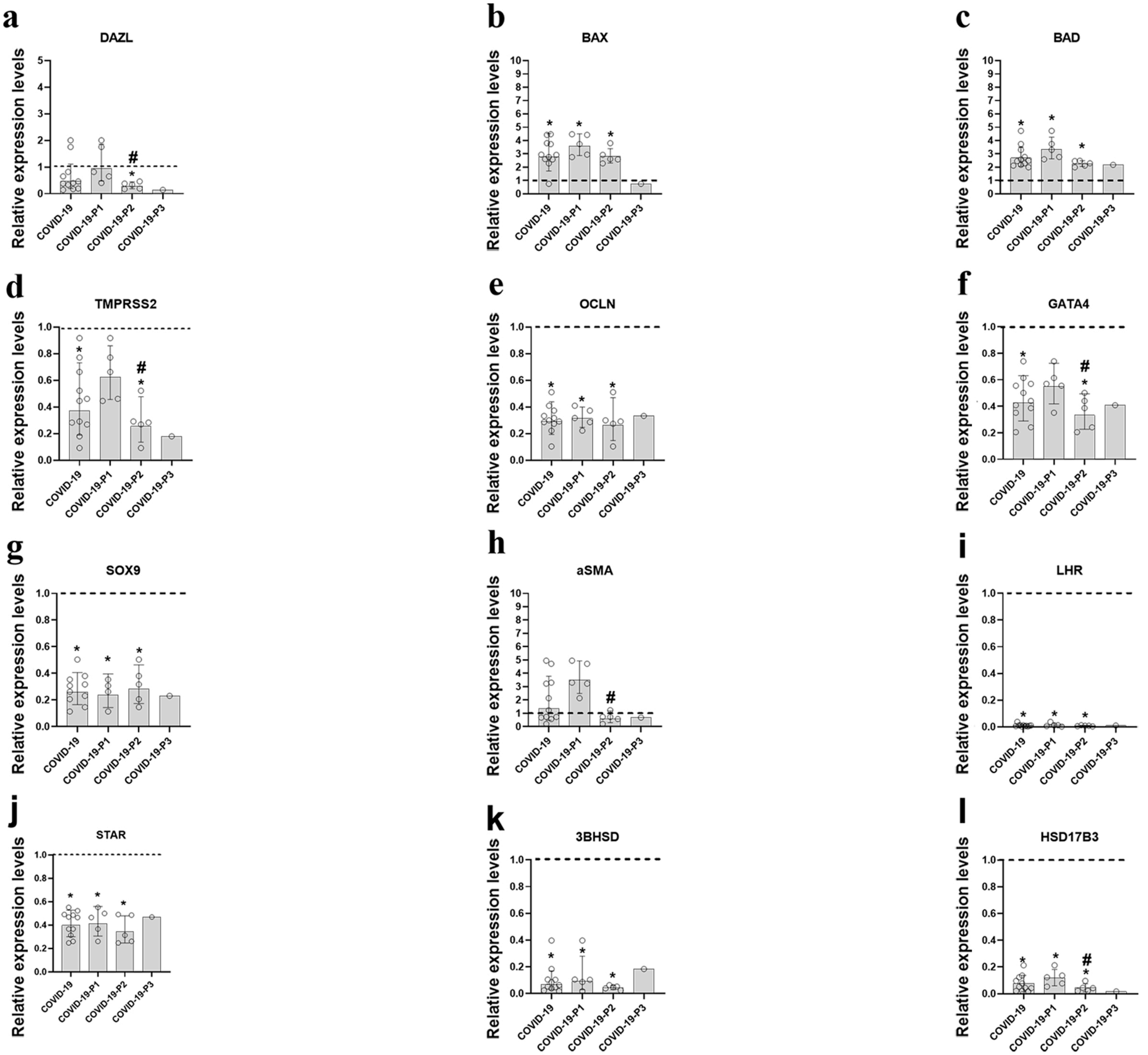
a
DAZL
b
BAX
c
BAD
d
TMPRSS2
e
OCLN
f
GATA4
g
SOX9
h
aSMA
i
LHR
j
STAR
k
3BHSD
l
HSD17B3
Relative expression levels
COVID-19
COVID-19-P1
COVID-19-P2
COVID-19-P3

Fig. S7. Relative expression of important genes associated with the tubular compartment and Leydig cells. a) Deleted In Azoospermia Like (DAZL). b) BCL2 Associated X Protein (BAX). c) BCL2 Associated Agonist Of Cell Death (BAD). d) Transmembrane Serine Protease 2 (TMPRSS2). e) Occludin (OCLN). f) GATA Binding Protein 4 (GATA4). g) SRY-Box Transcription Factor 9 (SOX9). h) Actin, Alpha Skeletal Muscle (aSMA). i-l) genes related to Leydig cell steroidogenesis: Luteinizing Hormone Receptor (i), Steroidogenic Acute Regulatory Protein (j), 3 Beta-Hydroxysteroid Dehydrogenase (k), Hydroxysteroid 17-Beta Dehydrogenase 3 (l). Data are expressed as geometric mean ± SD. *$p < 0.05$ COVID-19 patients compared to the Control group (which is set at 1; dashed line). # indicates significant differences between COVID-19-P1 and COVID-19-P2 ($p < 0.05$); t-test (two-sided). Individual values can be found at: https://doi.org/10.6084/m9.figshare.16777786.v8.
