# Supplementary material for: High SARS-CoV-2 tropism and activation of immune cells in the testes of non-vaccinated deceased COVID-19 patients
Source: BMC Biol. 2023 Feb 16;21:36. doi: 10.1186/s12915-022-01497-8 (PMC9933832; doi:10.1186/s12915-022-01497-8)
Supplement: Supplementary file 2 — Additional file 2: Table S1. Clinical data from control patients. Table S2. Antibodies and Primers (qPCR) used in this study. [file 12915_2022_1497_MOESM2_ESM.pdf]

Table S1. Clinical data from control patients.

Control Patient	Age	Fertility record (children)	FSH (mUI/ML)	LH (mUI/ML)	Testosterone (ng/dL)	Comorbidity
**#1**	62	Yes	2.57	3.91	227	Prostate cancer
**#2**	60	Yes	9.44	4.42	398	Prostate cancer
**#3**	58	Yes	1.49	4,18	523	Prostate cancer
**#4**	46	Yes	1.64	3.83	646	Prostate cancer
**#5**	57	Yes	2.41	3.68	288	Prostate cancer
**#6**	65	Yes	2.38	9.40	267	Prostate cancer
**Value of Reference**	-	-	1.55-9.74	0.82-6.22	71.8-623	-

Table S2. Antibodies and Primers (qPCR) used in this study.

Antibodies	Type	Species	Source	Id	Dilution
3βHSD	Polyclonal	Goat	Santa Cruz Biotech.	sc-30820	1:100
AGT1R	Polyclonal	Rabbit	Rhea Biotech	IM-0064	1:50
Anti- Protein S COVID-19	Monoclonal	Rabbit	Rhea Biotech	IM-0828	1:50
Anti-Spike RBD	Monoclonal	Mouse	Abcam	Ab277624	1:100
Caspase 3	Polyclonal	Rabbit	Imuny	IM0035	1:100
Caspase 8	Polyclonal	Mouse	Cell Signaling	Asp 387	1:100
CD68	Monoclonal	Mouse	Abcam	ab955	1:20
ECA2	Monoclonal	Mouse	Proteintech	AG15554	1:100
Endothelin	Polyclonal	Rabbit	Santa Cruz Biotech.	sc-98727	1:100
Mast Cell Chymase	Polyclonal	Goat	Santa Cruz Biotech.	sc-324924	1:100
TLR2	Polyclonal	Rabbit	Rhea Biotech	IM-0071	1:100
UTF1	Monoclonal	Mouse	EMD Millipore Corp.	Mab4337	1:100
Goat anti-rabbit (IgG-CFL 488)	Secondary	Goat	Santa Cruz Biotech.	sc-362262	1:200
Goat anti-mouse (IgG-488)	Secondary	Goat	Invitrogen	A-11001	1:1000
Goat anti-mouse (IgG- 546)	Secondary	Goat	Invitrogen	A-11003	1:200
Rabbit anti-goat (Cy3)	Secondary	Rabbit	Sigma	c2821	1:200
Goat anti-mouse (IgG-594)	Secondary	Goat	Jackson Immunoresearch	156816	1:100

Gene	Sequence Of Primer (5'-3')
3BHSD	F: CACATGGCCCGCTCCATAC
3β-Hydroxysteroid Dehydrogenase	R: GTGCCGCCGTTTTTCAGATTC
ACE1	F: CCA CGT CCC GGA AAT ATG AAG
Angiotensin I Converting Enzyme	R: AGT CCC CTG CAT CTA CAT AGC
ACE2	F: CAA GAG CAA ACG GTT GAA CAC
Angiotensin-Converting Enzyme 2	R: CCA GAG CCT CTC ATT GTA GTC T
AGTR1	F: ATTTAGCACTGGCTGACTTATGC
Angiotensin II Receptor Type 1	R: CAGCGGTATTCCATAGCTGTG
aSMA	F: CCC AGC CAA GCA CTG TCA
α-Smooth Muscle Actin	R: TCC AGA GTC CAG CAC GAT G
BAD	F: CCC AGA GTT TGA GCC GAG TG
BCL2 Associated Agonist of Cell Death	R: CCC ATC CCT TCG TCG TCC T
BAX	F: GAT GCG TCC ACC AAG AAG C
BCL2-associated X protein	R: CCA GTT GAA GTT GCC GTC AG
CASP3	F: GAC TGT GGC ATT GAG ACA GAC
Caspase-3	R: CTT TCG GTT AAC CCG GGT AAG

CLDN11<br>*Claudin 11*	F: CGGTGTGGCTAAGTACAGGC<br>R: CGCAGTGTAGTAGAAACGGTTTT
CMA1<br>*Chymase 1*	F: GGCTTCAACACACCTGTTCTT<br>R: TGGAAAACCACATTTGTGACGC
COX2<br>*Cyclooxygenase-2*	F: CTGGCGCTCAGCCATACAG<br>R: CGCACTTATACTGGTCAAATCCC
CX43<br>*Connexin 43*	F: GGT CTG AGT GCC TGA ACT TGC CT<br>R: TGC CTG GGC ACC ACT CTT TTG C
DAZL<br>*Deleted in azoospermia-like*	F: GCCCACAACCACGATGAATC<br>R: CGGAGGTACAACATAGCTCCTTT
GATA4<br>*GATA Binding Protein 4*	F: CCT GGC CTG TCA TCT CAC TAC<br>R: AGA GGA CAG GGT GGA TGG A
HPRT1<br>*Hypoxanthine Phosphoribosyltransferase 1*	F: CCT GGC GTC GTG ATT AGT GAT<br>R: AGA CGT TCA GTC CTG TCC ATA A
HRH4<br>*Histamine Receptor H4*	F: TGGGCCAATGATTCTAGTTTCAG<br>R: ACTAAGATGACTGGGATCACGAA
HSD17B3<br>*17β-Hydroxysteroid Dehydrogenase 3*	F: CCC ATC TAT TCG GTT CGT ATG GGC<br>R: GCC AGA GTC AGC GAA GGC GA
LHR<br>*Luteinizing Hormone Receptor*	F: GCT GTG CTT TTA GAA ACT TGC CAA CAA<br>R: TTC ATA GTC CCA GCC ACT CAG TTC ACT
MCP1<br>*Monocyte Chemoattractant Protein-1*	F: GTG TTC AAG TCT TCG GAG TT<br>R: CAA TAG GAA GAT CTC AGT GC
OCLN<br>*Occludin*	F: ACAAGCGGTTTTATCCAGAGTC<br>R: GTCATCCACAGGCGAAGTTAAT
PDGF<br>*Platelet-derived growth factor*	F: GCT GCT GCA ACA CGA GCA GT<br>R: CCG GAT TCA GGC TTG TGG TC
RPL19<br>*Ribosomal Protein L19*	F: GCG GGC CAA GGT GTT TTT C<br>R: TCG CCT CTA GTG TCC TCC G
SOX9<br>*SRY-Box Transcription Factor 9*	F: GAC TTC CGC GAC GTG GAC<br>R: GTT GGG CGG CAG GTA CTG
STAR<br>*Steroidogenic Acute Regulatory Protein*	F: GGGAGTGGAACCCCAATGTC<br>R: CCAGCTCGTGAGTAATGAATGT
TGFB1<br>*Transforming growth factor beta 1*	F: CTA ATG GTG GAA ACC CAC AAC G<br>R: TAT CGC CAG GAA TTG TTG CTG
TMPRSS2<br>*Transmembrane Serine Protease 2*	F: ACTCTGGAAGTTCATGGGCAG<br>R: TGAAGTTTGGTCCGTAGAGGC
TRPB2<br>*Tryptophan synthase beta chain 2*	F: TGGAAAACCACATTTGTGACGC<br>R: GACACGGGTGTAGATGCCA
VEGF<br>*Vascular endothelial growth factor*	F: CAC CCA TGG CAG AAG GAG GA<br>R: ACT CCA GGC CCT CGT CAT TG
